# Quasi-1D Chain-Based Zirconium Trisulfide as a Low-Potential High-Rate Anode: Structural and Reaction Mechanism Insights

**DOI:** 10.1021/acsami.5c22469

**Published:** 2026-04-16

**Authors:** Shuangying Wei, Min Liu, Ruizhi Yu, Haoyang Jiang, Huaijuan Zhou, Heng Li, Min Li, Payal Chauhan, Bing Wu, Takeshi Matsuo, Kseniia Mosina, Lukas Dekanovsky, Filipa M. Oliveira, Jan Luxa, Ondrej Jankovsky, Jincang Su, Zdeněk Sofer

**Affiliations:** † Department of Inorganic Chemistry, 52735 University of Chemistry and Technology Prague, Technická 5, Prague 6, 16628, Czech Republic; ‡ College of New Energy, 165069 Ningbo University of Technology, Ningbo, Zhejiang 315336, China; § Institute of Micro/Nano Materials and Devices, Ningbo University of Technology, Ningbo, Zhejiang 315211, China; ∥ School of Materials Science and Engineering, 12665 Xiangtan University, Xiangtan 411105, China; ⊥ Advanced Research Institute of Multidisciplinary Sciences, 47833 Beijing Institute of Technology, Beijing 100081, China; # School of Physics, Xi’an Jiaotong University, Xi’an 710049, China; ∇ Department of Mechanical Engineering, Okayama University, 3-cho̅me-1 Tsushimanaka, Kita Ward, Okayama 700-8530, Japan

**Keywords:** Quasi-1D materials, Zirconium trisulfide, Electrochemical reaction mechanisms, Structural evolution, Lithium-ion batteries

## Abstract

Transition metal trichalcogenides (TMTCs) of Group IVB (*e.g.*, ZrS_3_) are promising lithium-ion battery (LIB) anodes owing to their tunable band gaps, anisotropic conductivity, and high specific capacities. Here, microsized ZrS_3_ with a quasi-1D chain-based structure and van der Waals stacked layers were synthesized *via* a simple solid-state reaction. Subsequently, the ZrS_3_ anode was evaluated across distinct voltage windows, the storage mechanism switched from intercalation (≥1.0 V) to conversion (down to 0.001 V). The ZrS_3_ electrode delivers a high capacity of 844 mAh g^–1^ at 50 mA g^–1^ after 40 cycles, with excellent rate capability (281 mAh g^–1^ at 3000 mA g^–1^) and outstanding cycling stability, maintaining 408 mAh g^–1^ over 2300 cycles at 3000 mA g^–1^. *Ex situ* XRD/SEM-EDX/XPS track phase and surface evolution, while EIS resolves interfacial charge-transfer/ion-transport kinetics. DFT reveals low-barrier Li^+^ diffusion along interchain pathways in bulk (≈0.12 eV) and monolayer ZrS_3_. A directional increase in the calculated Young’s modulus under small strain suggests robust mechanics upon cycling. These experimental–theoretical insights establish ZrS_3_ as a low-potential, high-rate anode for lithium-ion batteries and clarify the intercalation–conversion crossover in Group IVB TMTCs.

## Graphical abstract

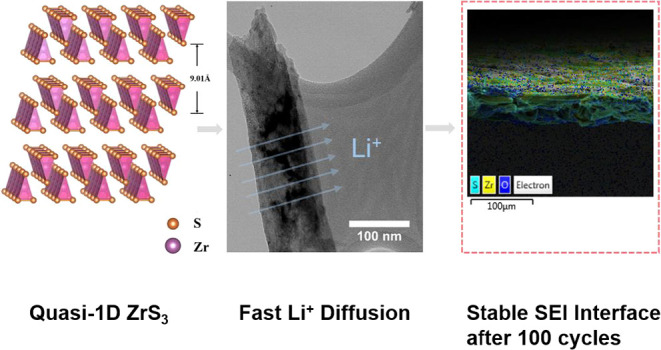

## 1 Introduction

Lithium-ion batteries (LIBs) are currently the most prevalent energy storage technology, widely applied in portable electronics and electric vehicles (EVs).^1^ Their widespread adoption stems from key advantages such as high energy density, long cycle life, efficient energy conversion, and low self-discharge rates.^2^ However, as the global demand for safer, more efficient, and high-performance energy storage solutions continues to grow, advancing LIB technology remains a critical priority.^3^ One of the most important performance parameters in LIBs is rate capability, which determines the ability to sustain high charge/discharge currents without significant capacity loss.^4^ This capability is influenced by multiple factors, including electronic and ionic transport, interface charge transfer, desolvation processes, electrode architecture and electrolyte composition. Among these, ionic diffusion within the bulk electrode is frequently a rate-limiting step.^5^ To address this limitation, extensive research has focused on nanostructured electrode designs that reduce ion diffusion distances and improve charge transport kinetics.^6^ However, such complex nanoarchitectures often come with trade-offs, including reduced volumetric energy density, structural instability, higher manufacturing costs, and environmental concerns. An alternative approach is to explore unique crystal structures that inherently facilitate fast Li^+^ insertion and extraction, thereby achieving both ultrahigh-rate capability and long-term stability.^7,8^

Recently, mixed titanium–niobium oxides (*e.g.*, TiNb_2_O_7_) have been explored as high-rate anode materials due to their three-dimensional (3D) interconnected tunnel structures that enable rapid Li^+^ diffusion.^9^ However, their relatively high operating potentials (>1.5 V *vs* Li^+^/Li) limit the full-cell voltage and energy density when paired with conventional cathodes. Thus, there is an urgent need to develop novel rigid host lattices that combine low operating potential with high-rate performance.^10^

Transition-metal chalcogenides (TMCs) have attracted considerable interest due to their tunable electronic structures, rich redox chemistry, and decent electronic conductivity.^11^ Although they were proposed early as electrodes for rechargeable LIBs, transition-metal trichalcogenides (TMTCs) have received comparatively little attention in this field.^12,13^ These materials display quasi-one-dimensional (quasi-1D) or layered structures, following the general formula M*X*_3_, where M represents a group IVB (Ti, Zr, Hf) or VB (Nb, Ta) transition metal, and *X* is a group VIA (S, Se, Te) chalcogen.^14^ Structurally, TMTCs consist of strong covalent bonds along 1D atomic chains, while adjacent chains are held together by van der Waals (vdW) interactions, imparting both quasi-1D and layered characteristics.^15^ The fundamental structural unit is a 1D chain of M*X*_3_ prisms, which gives rise to distinct electronic and mechanical properties.^16^ The physical properties of M*X*_3_ compounds span a broad range, from superconducting to semiconducting, and even highly insulating, depending on the stacking sequence and interchain interactions.^17^

Among these materials, zirconium trisulfide (ZrS_3_) has been less explored, and systematic electrochemical studies remain scarce.^18^ The structural and transport properties of ZrS_3_ were first extensively studied by Professor Jean Rouxel in the late 1980s.^19^ His pioneering work laid the foundation for understanding the quasi-1D channel structure of ZrS_3_, which was recognized as a key feature enabling enhanced ion diffusion and electrochemical stability.^20^ ZrS_3_ exhibits a unique layered structure, in which vdW interactions between layers facilitate efficient Li-ion intercalation and diffusion.^21^ Unlike many TMDC anodes (*e.g.*, MoS_2_, WS_2_), which utilize interlayer galleries for Li^+^ storage,^22^ ZrS_3_ integrates both chain-like and layered motifs, providing additional Li-ion storage sites.^23^ Furthermore, at low voltages, ZrS_3_ undergoes a conversion reaction, providing additional capacity, albeit often with trade-offs in structural stability.^24^ Despite these advantages, the reaction mechanisms and electrochemical behavior of ZrS_3_ have remained largely unexplored both experimentally and theoretically.

Herein, microsized quasi-1D ZrS_3_ microbelts were prepared through a facile solid-state reaction and their morphology and structural properties were systematically examined using XRD, SEM, TEM, and XPS. We evaluated the electrochemical performance of ZrS_3_ as an anode material for LIBs, analyzing how its reaction mechanisms shift between intercalation and conversion processes at different cutoff voltages (1.0, 0.3, and 0.001 V). Even without carbon decoration or nanostructuring, microsized ZrS_3_ demonstrates excellent lithium storage properties, including high specific capacity, superior rate capability, and long-cycle stability. Morphological and compositional changes were monitored using *ex-situ* XRD, SEM, SEM-EDX, cross-section SEM, and XPS. Additionally, DFT calculations revealed that Li-ion diffusion in bulk ZrS_3_ follows a pathway between adjacent stable H sites across T sites, with a relatively low energy barrier of 0.12 eV, enabling fast Li-ion transport and high charge–discharge rates. DFT predicts direction-dependent stiffening upon lithiation: in each of the three principal planes (*xz*, *yz*, and *xy*), the Young’s modulus of LiZrS_3_ exceeds that of pristine ZrS_3_ across the entire angular range within the linear-elastic regime.

## 2 Discussion

ZrS_3_ is a typical transition metal trisulfide, with its structure consisting of both disulfide (S_2_^2–^) and sulfide (S^2–^) anions.^25^ The fundamental building blocks of ZrS_3_ are 1D chains, where Zr^4+^ cations are coordinated with these anions.^26^ These chains are covalently bonded along their lengths, forming stable 1D structures. Through weak van der Waals-like interactions, these 1D chains assemble into 2D layers,^27^ characterized by an interlayer spacing of 9.01 Å, as depicted in Figure 1a. The 2D layers then stack through additional van der Waals interactions, resulting in a bulk crystal with a prismatic morphology. This unique stacking mechanism results in a layered structure, where the interlayer forces are significantly weaker than the covalent bonds within the chains.^28^

**Figure 1:**
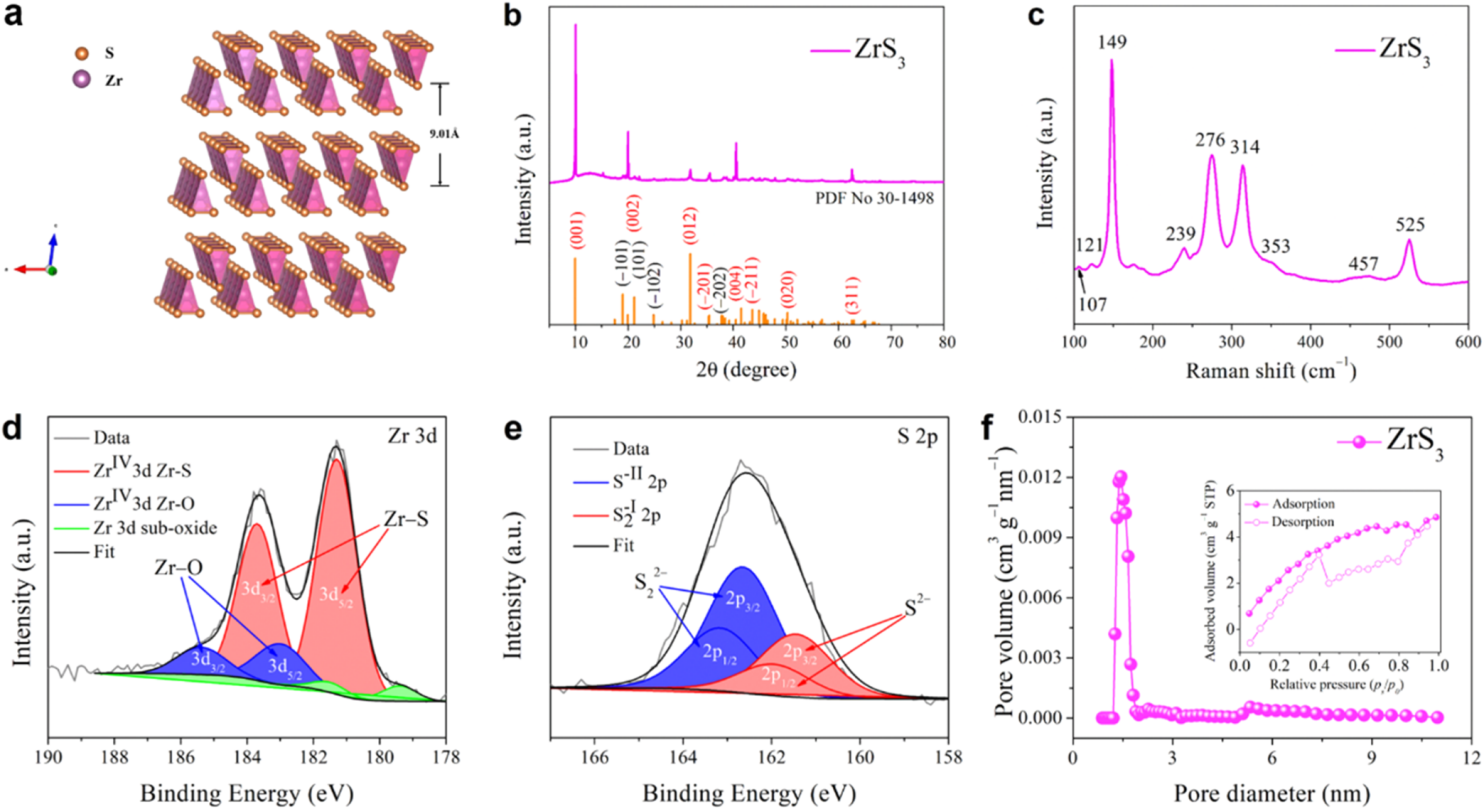
(a) Schematic illustration of the crystal structure of ZrS_3_. (b) XRD pattern of ZrS_3_. (c) Raman spectra of ZrS_3_. High-resolution XPS spectra of ZrS_3_, (d) Zr 3d, (e) S 2p core levels. (f) Pore size distribution and N_2_ adsorption–desorption isotherm (insert) curve of ZrS_3_.

A thorough set of material characterizations was carried out to understand the structural, chemical, and morphological properties of ZrS_3_. The powder XRD pattern (Figure 1b) confirms the successful synthesis of phase-pure ZrS_3_ (JCPDS No. 30–1498), which crystallizes in a monoclinic structure with the space group *P*2_1_/*m*.^20^ Notably, no discernible impurity peaks were observed, indicating the high purity of the material. The characteristic diffraction peaks observed at 10.13°, 19.86°, 31.82°, 35.41°, 40.68°, 43.6°, 50.30°, and 62.49° correspond to the (001), (002), (012), (−201), (004), (−211), (020) and (311) planes, respectively. The Raman spectrum for ZrS_3_ (Figure 1c) exhibits four active Raman modes at 149, 276, 314, and 525 cm^–1^. The pronounced Raman peak at 525 cm^–1^ is attributed to the stretching mode of the (S–S)^2–^ group.^29^ Additionally, a peak at 457 cm^–1^ is considered to be a combination peak, while the peak at 353 cm^–1^ is attributed to second-order scattering effect.^30^ XPS confirms the chemical composition and oxidation states of the elements present in ZrS_3_. The high-resolution Zr 3d XPS spectra (Figure 1d) reveal two prominent peaks at 181.3 ± 0.1 eV and 183.7 ± 0.1 eV, which correspond to Zr^4+^.^31^ The low spectrum displays the Zr 3d_3/2_ and Zr 3d_5/2_ peaks of ZrO_*x*_, located at 183.0 ± 0.1 eV and 185.4 ± 0.1 eV, respectively. The spin–orbit doublet splittings for ZrS_3_ and ZrO_*x*_ are 2.4 eV,^32^ confirming the presence of a thin surface oxide layer on the ZrS_3_ microbelts. Similarly, the XPS S 2p spectra (Figure 1e) reveal the coexistence of sulfide (S^2–^) and disulfide (S_2_^2–^) species, fitted using two distinct doublet peaks. The binding energies of the S 2p_1/2_ and S 2p_3/2_ peaks for sulfide are 162.3 ± 0.1 eV and 162.9 ± 0.1 eV, respectively, whereas those for the disulfide group appear at 163.4 ± 0.1 eV and 163.9 ± 0.1 eV.^33^ The textural properties of ZrS_3_ were analyzed using the Brunauer–Emmett–Teller (BET) method, revealing a specific surface area of 3.505 m^2^ g^–1^, a total pore volume of 0.0065 cm^3^ g^–1^, and an average pore size of 4.038 nm (Table S1, Figure 1f). The BET results indicate a relatively low specific surface area (3.505 m^2^ g^–1^) and limited pore volume (0.0065 cm^3^ g^–1^), suggesting a compact and dense morphology, typical of bulk layered sulfides. Nevertheless, the observed average pore size of 4.038 nm falls within the mesoporous range (2–50 nm), implying the presence of a limited number of mesopores.

The SEM images in Figure 2a exhibit a well-defined belt-like structure with smooth surfaces and a layered morphology. These microbelts have relatively high aspect ratios, likely due to the intrinsic anisotropic growth of ZrS_3_ crystals along the quasi-1D direction.^34,35^ The belt length ranges from 15 to 50 μm, with thicknesses varying between 1 to 10 μm. The SEM-EDX analysis confirms the uniform distribution of Zr and S, as depicted in Figure 2a. Upon further investigation using TEM, the microbelt structure transforms into exfoliated ZrS_3_ nanoribbons. This transition can be attributed to the weak vdW interactions between adjacent layers, which makes ZrS_3_ highly susceptible to exfoliation under ultrasonication or sample preparation processes.^21^

**Figure 2:**
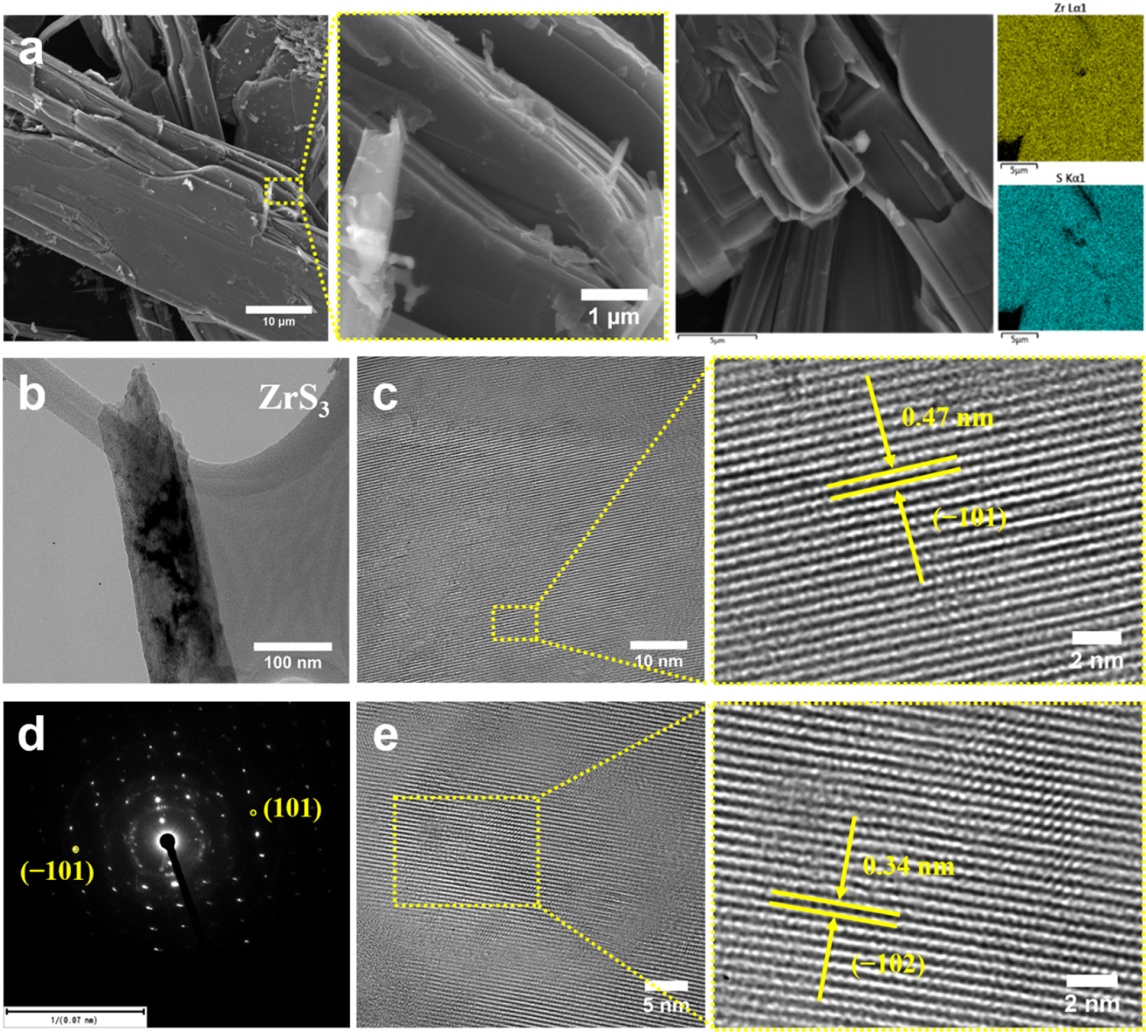
(a) SEM images of ZrS_3_ at different magnifications with corresponding EDX mappings. (b) TEM, (c, e) HRTEM images, and (d) SAED pattern of a single-crystalline ZrS_3_ nanoribbon.

The TEM image (Figure 2b) reveals the strip-like morphology of an exfoliated ZrS_3_ nanoribbon, where regions of varying contrast correspond to differences in thickness. The high-resolution TEM (HRTEM) images (Figure 2c and e) further confirm the well-defined lattice fringes of ZrS_3_ nanoribbons. The measured interplanar spacing from Figure 2c is approximately 0.47 nm, which matches well with the (−101) plane of monoclinic ZrS_3_ (JCPDS No. 30–1498, *d* = 0.4677 nm). In contrast, Figure 2e shows a spacing of 0.34 nm, corresponding to the (−102) plane (*d* = 0.3582 nm). These observations reveal the high crystallinity of the synthesized material and the presence of multiple exposed facets. Additionally, the selected area electron diffraction (SAED) pattern (Figure 2d) exhibits a well-defined set of diffraction spots, which can be indexed to the monoclinic ZrS_3_ crystal structure with the major reflections labeled, indicating the single-crystalline nature of the nanoribbon. These multiscale structural characterizations confirm the successful synthesis of high-quality layered ZrS_3_ with a quasi-1D building unit and ribbon-like morphology.

The electrochemical insertion/extraction and conversion mechanisms of the ZrS_3_ electrode were initially investigated through cyclic voltammetry (CV) measurements at a scan rate of 0.2 mV s^–1^ over the first three cycles, exploring different voltage windows of 1.0–3.0 V, 0.3–3.0 V, and 0.001–3.0 V, respectively (Figure 3a–c). The cathodic and anodic peaks in the CV results indicate multistep intercalation and conversion reactions. Specifically, these processes involve the insertion of Li^+^ into the ZrS_3_ layered structure, leading to changes in the sulfur electronic environment and the reduction of S_2_^2–^ to S^2–^.^36^ It is important to note that during the first scan across voltage range of 1.0–3.0 V (Figure 3a), two key electrochemical characteristics are observed: (i) a reduction peak near 1.8 V and (ii) a corresponding oxidation peak near 2.0 V. These peaks are indicative of the reversible Li^+^ intercalation and extraction within the ZrS_3_ structure, as described by eq 1.

1 $${\mathrm{ZrS}}_{3}+{x\mathrm{Li}}^{+}+xe^{-}↔{\mathrm{Li}}_{x}{\mathrm{ZrS}}_{3}$$

**Figure 3:**
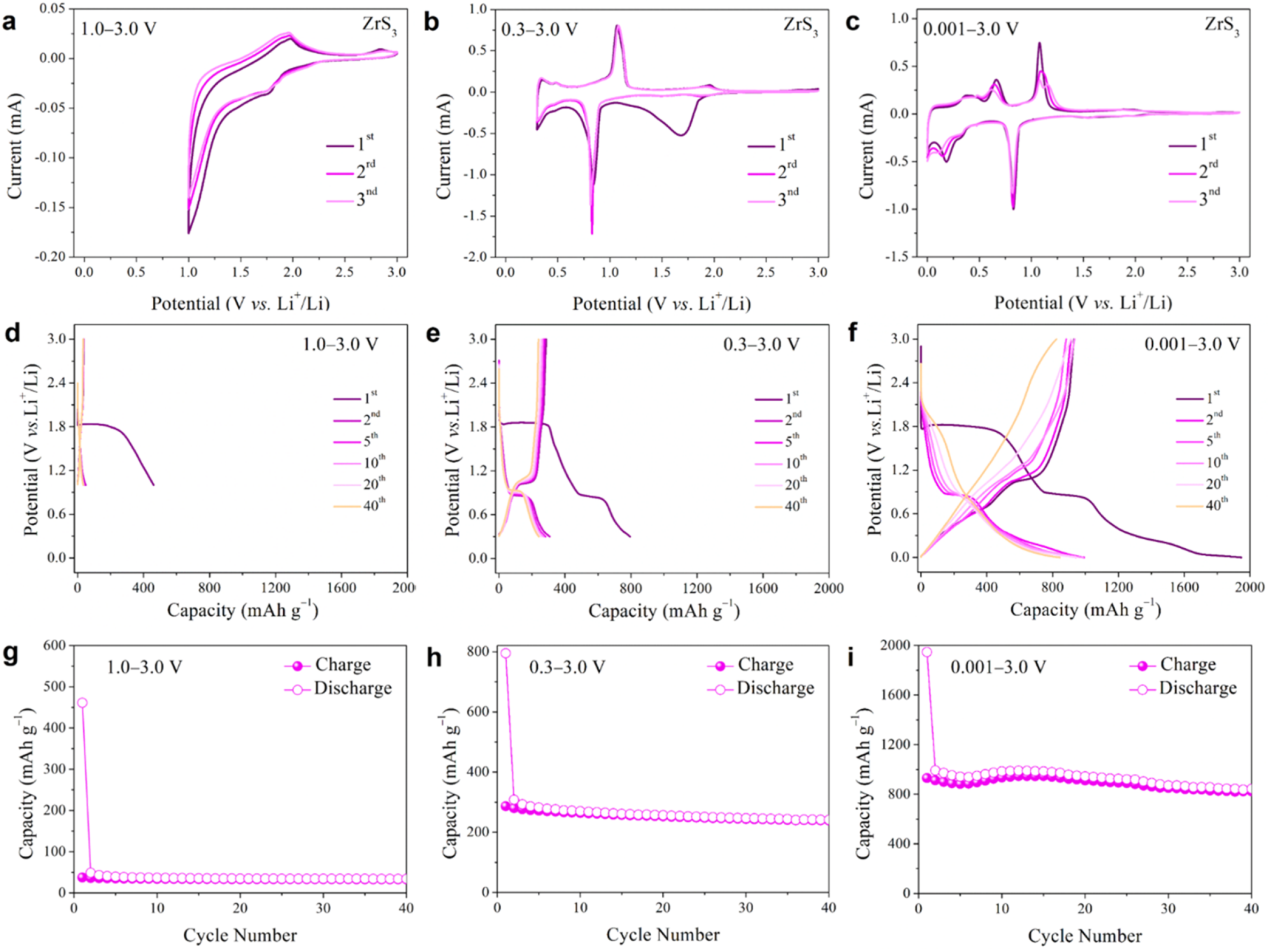
Electrochemical performance evaluation of ZrS_3_ electrodes in lithium coin cells in the voltage ranges of 1.0–3.0 V, 0.3–3.0 V, and 0.001–3.0 V. (a–c) Cyclic voltammetry curves at a scan rate of 0.2 mV s^–1^. (d–f) Charge/discharge profiles for selected cycles at a current density of 50 mA g^–1^. (g–i) Cycling performance over 40 cycles.

The slight separation between the reduction and oxidation peaks is attributed to polarization, and the two distinct plateaus in the charge/discharge curves (Figure 3d) correspond to phase transitions during Li^+^ intercalation/extraction.^37^

For the voltage window of 0.3 to 3.0 V, multiple oxidation peaks appear during the initial anodic sweep (Figure 3b), reflecting the stepwise nature of the conversion process with partial reversibility, as shown by eq 2.

2 $${\mathrm{Li}}_{x}{\mathrm{ZrS}}_{3}+\left(6-x\right){\mathrm{Li}}^{+}+\left(6-x\right)e^{-}\to 3{\mathrm{Li}}_{2}S+\mathrm{Zr}$$

As the depth of discharge increases in the first cycle, peaks appear around 0.8 V, which are primarily associated with conversion reactions,^38^ while SEI formation occurs at lower voltages (<0.5 V).^39^ In the anodic scan, peaks near 1.1 V can be attributed to partial delithiation and Zr–S bond reorganization within the electrode, rather than full Li_2_S oxidation, indicating that the ZrS_3_ structure can partially recover during cycling. It should be noted that complete Li_2_S oxidation typically occurs at higher voltages (∼2.0–2.5 V).^40^ In subsequent scans of the voltage window of 0.3 to 3.0 V, the CV curves exhibit well-defined cathodic and anodic peaks, suggesting a moderate degree of reversibility in the phase transformations during lithiation/delithiation. For the voltage window of 0.001 to 3.0 V (Figure 3c), as the depth of discharge increases, peaks appear around 0.2 V, which are associated with deep conversion reactions.^41^ In the anodic scan, peaks near 0.6 V may be associated with partial oxidation of Li_2_S-related intermediates, contributing to some reversibility.^38^ Additionally, the persistence of these peaks in subsequent cycles suggests a certain degree of reversibility for the insertion/extraction and conversion processes, though capacity fading may still occur due to SEI growth and incomplete conversion.^42^

To elucidate the electrochemical mechanisms of ZrS_3_ electrodes, galvanostatic discharge/charge measurements were conducted across three voltage ranges: 1.0–3.0 V, 0.3–3.0 V, and 0.001–3.0 V (Figure 3d–f, 3g–i). In the 1.0–3.0 V range, as shown in Figure 3d, lithium intercalation is the dominant mechanism, with initial discharge and charge capacities of 461 mAh g^–1^ and 37.4 mAh g^–1^, respectively. The significant irreversible capacity loss, likely due to SEI formation, irreversible side reactions and the limited reversibility of Li^+^ ion extraction, results in a low initial Coulombic efficiency (ICE) of 8.1%.^43^ During subsequent cycles, the ZrS_3_ electrode exhibits minor capacity fade, maintaining a discharge capacity of 33.5 mAh g^–1^ after 40 cycles (Figure 3g). This fade may be attributed to electrolyte decomposition, SEI stabilization, and persistent side reactions between Li^+^ and ZrS_3_.^44,45^ These findings indicate that the 1.0–3.0 V range primarily supports intercalation reactions, contributing to stable but modest capacity retention during cycling. In the 0.3–3.0 V voltage range, both intercalation and partial conversion reactions contribute to the electrochemical performance of the ZrS_3_ electrode. As shown in Figure 3e, the initial discharge and charge capacities reach 795 mAh g^–1^ and 287 mAh g^–1^, with 36.1% ICE, which is notably higher than that of the 1.0–3.0 V range. A discharge plateau at 0.3 V is observed, suggesting that partial conversion reactions occur with moderate reversibility. However, structural changes and gradual electrode reorganization may still lead to capacity fading over cycling, as confirmed by Figure 3h. This voltage range allows for deeper lithiation, initiating a partial conversion reaction, where ZrS_3_ begins decomposing into metallic Zr and Li_2_S, while metallic Zr mainly remains electrochemically inactive with respect to Li storage within the investigated potential window, though without complete conversion.^46^ This partial reaction provides additional lithium storage sites beyond those accessible by intercalation alone, yielding a discharge capacity of 241 mAh g^–1^ after 40 cycles. Importantly, because complete conversion is avoided, the structural stability of the electrode is largely preserved. This range thus benefits from both the reversible intercalation process and partial conversion reactions, achieving a balance of increased capacity and structural integrity. In the 0.001–3.0 V voltage range, both intercalation and conversion reactions dominate, resulting in high initial discharge and charge capacities of 1946 mAh g^–1^ and 930 mAh g^–1^, with an ICE of 47.8%. The sustained discharge plateau at 0.3 V in subsequent cycles highlights the partial reversibility of conversion reactions, as observed in Figure 3f. The ZrS_3_ electrode retains a discharge capacity of 844 mAh g^–1^ after 40 cycles (Figure 3i), reflecting moderate capacity retention, though some degradation may result from the irreversible nature of the conversion reaction and incomplete reoxidation of Li_2_S.^47^

To investigate the structural evolution of ZrS_3_ during cycling, *ex-situ* XRD measurements were performed at different voltage windows (1.0–3.0 V, 0.3–3.0 V, and 0.001–3.0 V), as shown in Figure S1. The results reveal voltage-dependent peak shifts and partial reversibility, indicating a predominant intercalation mechanism with limited conversion reactions (Details are provided in the Supporting Information). The in-plane polar diagram of the Young’s modulus plots of bulk ZrS_3_ before and after lithiation is illustrated in Figure S2. The results indicate that the Young’s modulus of lithiated LiZrS_3_ is significantly higher than that of pristine bulk ZrS_3_ in all directions, implying enhanced mechanical stability during lithium intercalation and deintercalation processes. By investigating this broader window, a more complete picture of the underlying mechanisms can be obtained, which is critical for optimizing ZrS_3_ as an anode material for LIBs. A detailed structural and electrochemical comparison of ZrS_3_ with TiS_3_ and NbS_3_ is provided in Table S2 (Supporting Information), highlighting its wider Li^+^ transport channels, weaker interlayer coupling, and lower discharge potential.

To investigate the morphological and compositional evolution of the ZrS_3_ electrode during cycling, *ex-situ* SEM, SEM-EDX, and cross-sectional SEM analyses were performed at different cycle numbers under varying current densities. The results for 0–100 cycles at 100 mA g^–1^ are displayed in Figures 4, S3, and Table S3. SEM micrographs at high magnifications reveal a relatively smooth electrode surface with no significant aggregation after 0 cycles (Figure 4a), 1 cycle (Figure 4b) and 10 cycles (Figure 4c). However, after 100 cycles (Figure 4d), the surface becomes rougher with small aggregated features, indicating gradual surface reconstruction. SEM-EDX mapping (Figure 4e–h) indicates a homogeneous distribution of O across the electrode surface. With increased cycling (10 and 100 cycles, Figure 4g and 4h), the oxygen signal intensifies, suggesting progressive accumulation of SEI components or partial surface oxidation, possibly forming ZrO_2_. This surface oxidation is limited to extent and is not expected to act as an active Li-storage phase; instead, it may contribute to interfacial stabilization by forming a passivation-like layer that suppresses continuous side reactions with the electrolyte during prolonged cycling. This oxidation process may originate from continuous exposure to trace moisture or residual oxygen in the electrolyte, and is commonly observed in sulfide-based electrodes.^48^ Cross-sectional SEM (Figure 4i–l) reveals that the electrode thickness initially increases from ∼46 μm to ∼65 μm, likely due to SEI formation and electrolyte infiltration, and then gradually decreases to ∼51 μm upon extended cycling. This trend indicates electrode densification and interfacial compaction over time, rather than irreversible expansion or mechanical pulverization. Such structural stabilization is beneficial for long-term cycling performance. Detailed observations, including surface roughening, partial oxidation, and electrode thickness changes, are summarized in (Figure S3 and S4, Supporting Information).

**Figure 4:**
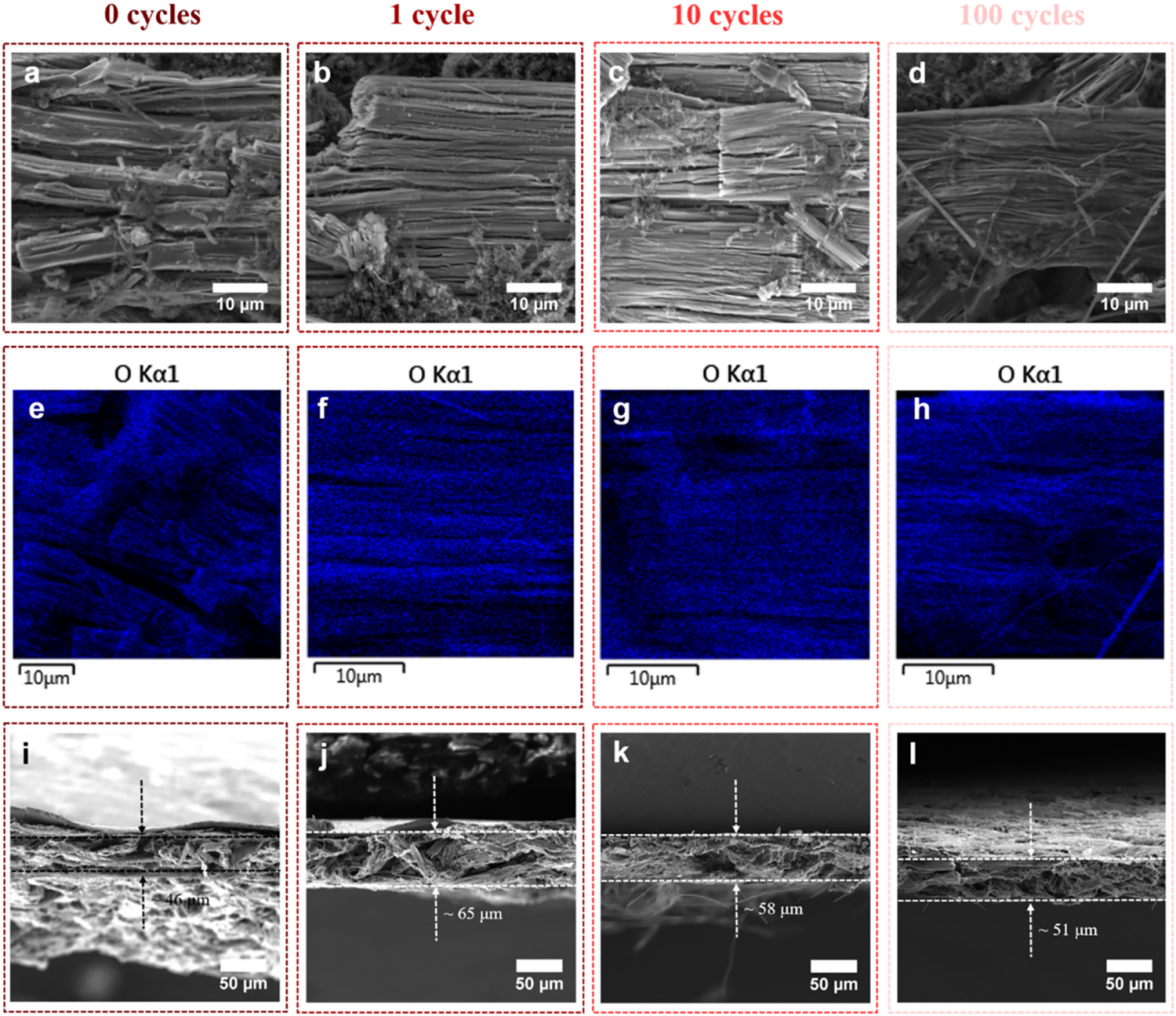
Morphological evolution of ZrS_3_ electrodes during cycling at 100 mA g^–1^ (0.001–3.0 V). (a–d) Top-view SEM images at high magnifications after 0, 1, 10, and 100 cycles. (e–h) O Kα elemental mappings after 0, 1, 10, and 100 cycles. (i–l) Cross-sectional SEM images after 0, 1, 10, and 100 cycles.

To further elucidate the structural evolution of ZrS_3_ during deep cycling, additional *ex situ* XRD measurements were conducted over a voltage range of 0.001 to 3.0 V across two full discharge/charge cycles, as illustrated in Figure 5a. By systematically tracking diffraction pattern changes at 13 distinct voltage states (A–L), we gain deeper insights into the interplay between intercalation and conversion reactions. The XRD analysis indicates that ZrS_3_ undergoes both intercalation and conversion reactions, consistent with reported behaviors of layered transition metal sulfides like ZrS_2_.^44^

**Figure 5:**
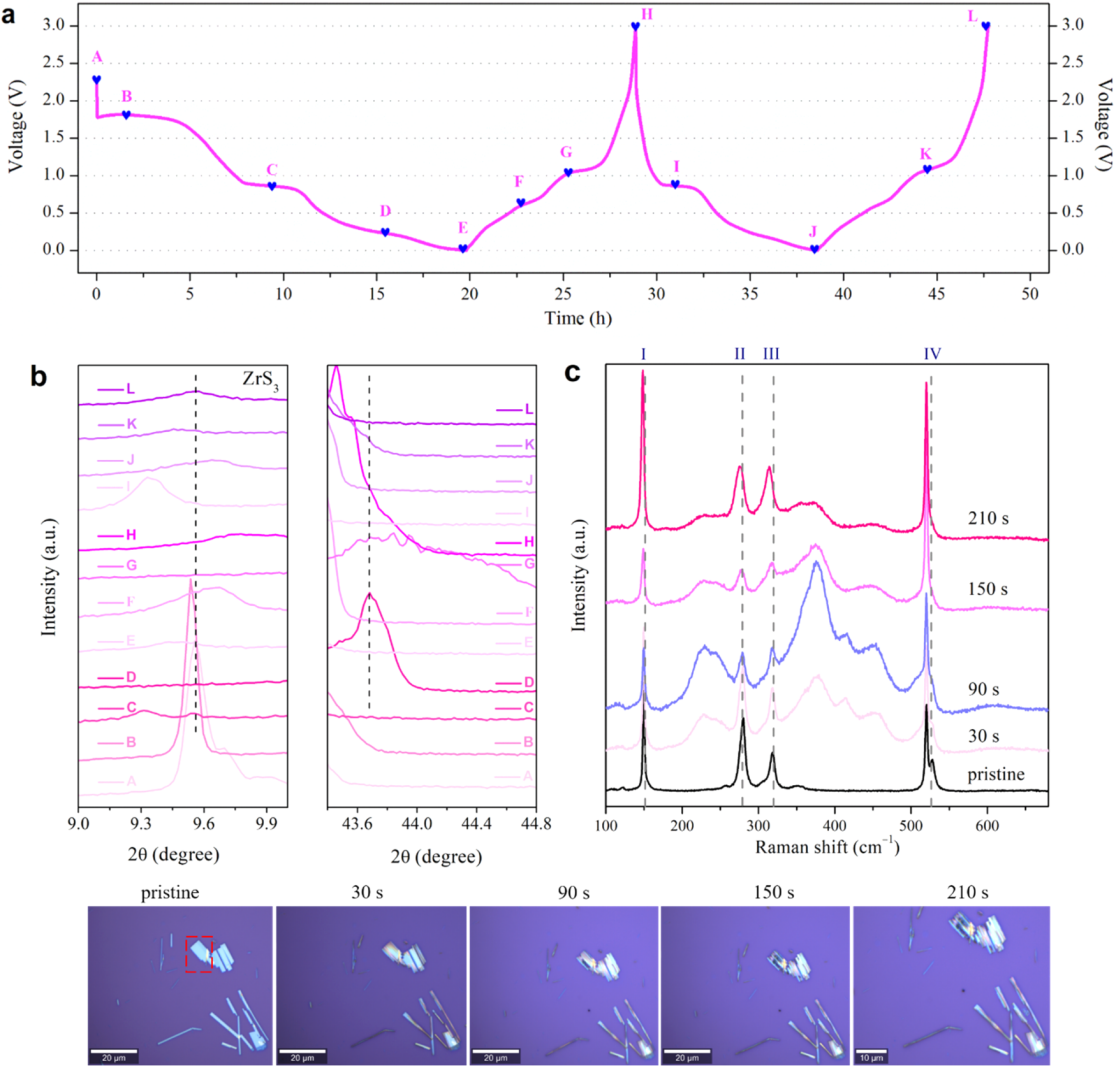
(a) Real-time galvanostatic discharge/charge profile of the ZrS_3_ electrode with labeled sampling points (A–L) marked for *ex situ* XRD analysis. (b) Zoomed-in *ex situ* XRD patterns of the ZrS_3_ electrode showing peak evolution at around 9.6° and 43.6°. (c) Time-resolved optical and Raman spectroscopy analysis of lithium intercalation in ZrS_3_ flakes.

As shown in Figure 5b, the diffraction peak at ∼9.6° corresponding to the (001) plane of ZrS_3_ remains nearly unchanged between the open-circuit voltage (OCV, state A) and 1.84 V (state B), indicating a lithium intercalation-dominated process with minimal structural distortion. Upon further discharge to 0.89 V (state C) and 0.23 V (state D), this peak gradually vanishes, signaling the onset of a conversion reaction that disrupts the layered framework. A new diffraction peak emerges at ∼43.6° (Figure 5b, right panel) during deep discharge (state D), which can be attributed to the formation of Li_2_S and/or metallic Zr, as these two phases exhibit overlapping reflections in this diffraction region.^44^ This peak fades during charging (states F–G) and is completely absent in the second-cycle stages (states I–L), highlighting the partial irreversibility of the conversion process. Upon recharging to 3.0 V (state H), the (001) peak at ∼ 9.6° partially reappears, suggesting partial reconstruction of the ZrS_3_ framework. However, the overall XRD pattern does not fully return to its initial state, evidencing incomplete structural reversibility. During the second cycle, XRD patterns recorded at 0.86 V (state I) and 0.001 V (state J) confirm partial lithiation and structural reformation. However, the intensities of the main peaks remain significantly altered compared to those in the pristine state. At 1.09 V (state K) and 3.0 V (state L), further lithium extraction improves crystallinity but still fails to fully restore the initial structure. These results collectively support a dual lithium storage mechanism involving both intercalation and conversion, with irreversible phase changes contributing to capacity fading. At lower discharge potentials, where the conversion reaction becomes dominant (states C–D), the loss of long-range order limits the applicability of Raman analysis, and the structural evolution is instead captured by *ex situ* XRD.

The time-resolved Raman spectroscopy primarily reflects the lithium intercalation process occurring at relatively higher potentials, where the ZrS_3_ layered framework is preserved, corresponding to the intercalation-dominated voltage region identified in the *ex-situ* XRD analysis (states A–B in Figure 5a). To further elucidate the lithium intercalation behavior of ZrS_3_ and validate its structural anisotropy, *in situ* optical microscopy and time-resolved Raman spectroscopy were performed. Bulk ZrS_3_ was mechanically exfoliated and stamped onto a Si/SiO_2_ substrate, which was repeatedly immersed into a 1 mM solution of lithium anthracenide for various time periods.^49^ Figure 5c displays the optical images captured during lithiation and the corresponding time-resolved Raman spectra. Since ZrS_3_ exhibits pronounced anisotropic properties, the Raman spectrum was acquired from a ZrS_3_ flake oriented at an approximate 45° angle, ensuring the presence of primary Raman modes. The Raman signal collected from the pristine ZrS_3_ flake marked with a red rectangle with a thickness of ca. 30 nm demonstrated four characteristic Ag vibrations at 150, 280, 318, and 528 cm^–1^. Upon intercalation, peaks started to shift to lower energy levels. Remarkably, we noticed that intercalation propagated exclusively along the a-direction, which is well seen in the images taken at 150 and 210 s of intercalation. After 210 s, three modes of pristine ZrS_3_ had shifted, namely, the peak I–Ag shifted by 1 cm^–1^ to a lower energy value of 149 cm^–1^, the peaks II–Ag and III–Ag were significantly widened and both shifted by 5 cm^–1^, while the peak IV–Ag had diminished. We attribute the different influence of intercalation on intercalated ZrS_3_ vibrational modes to the quasi-one-dimensional nature of this crystal. The I–Ag vibration is dominated by the interlayer interaction, while III–Ag and IV–Ag are affected by both the inter- and intralayer interactions,^50^ which is reflected in the shift of these modes upon intercalation due to the crystal lattice expansion. The observed phonon behavior thus reflects lattice expansion and vibrational perturbation along specific crystallographic directions, providing additional vibrational evidence for the directional intercalation process proposed in the reaction mechanism.

To thoroughly evaluate the high-rate electrochemical performance of the ZrS_3_ electrode, a systematic investigation was conducted across a range of high current densities, as shown in Figure 6. The electrode was initially activated at a low current density of 25 mA g^–1^, facilitating complete lithiation and stabilizing the electrode structure. Following this activation, the current density was sequentially increased to 1500, 2000, 2500, and 3000 mA g^–1^. After reaching the peak current density, the current was progressively reduced back to 1500 mA g^–1^. Figure 6a presents the galvanostatic charge/discharge potential profiles of the ZrS_3_ electrode at selected cycles. The electrode delivered an initial discharge capacity of 415 mAh g^–1^, with an ICE of 83.8%, partly attributed to irreversible activation processes. The fifth charge/discharge profile shows overlapping curves in subsequent cycles, demonstrating the high reversibility and structural stability of the lithium storage reaction throughout extended cycling. The ZrS_3_ electrode displays high discharge capacities of 329, 311, 295, and 281 mAh g^–1^ at current densities of 1500, 2000, 2500, and 3000 mA g^–1^, respectively (Figure 6b). Upon returning to 1500 mA g^–1^, the capacity recovered to 338 mAh g^–1^, highlighting both excellent rate capability and structural integrity. To further evaluate the rate response of the electrode, a nonreversible rate test (without current return) was conducted from 25 to 3000 mA g^–1^ (Figure S6). Rate-dependent impedance spectra collected after 10 cycles (Figure S7) revealed progressive increases in interfacial resistance (*R*_SEI_ and *R*_ct_) with increasing current, indicating kinetic limitations at high rates. The corresponding fit parameters are summarized in Table 4, based on the equivalent circuit shown in Figure S7c.

**Figure 6:**
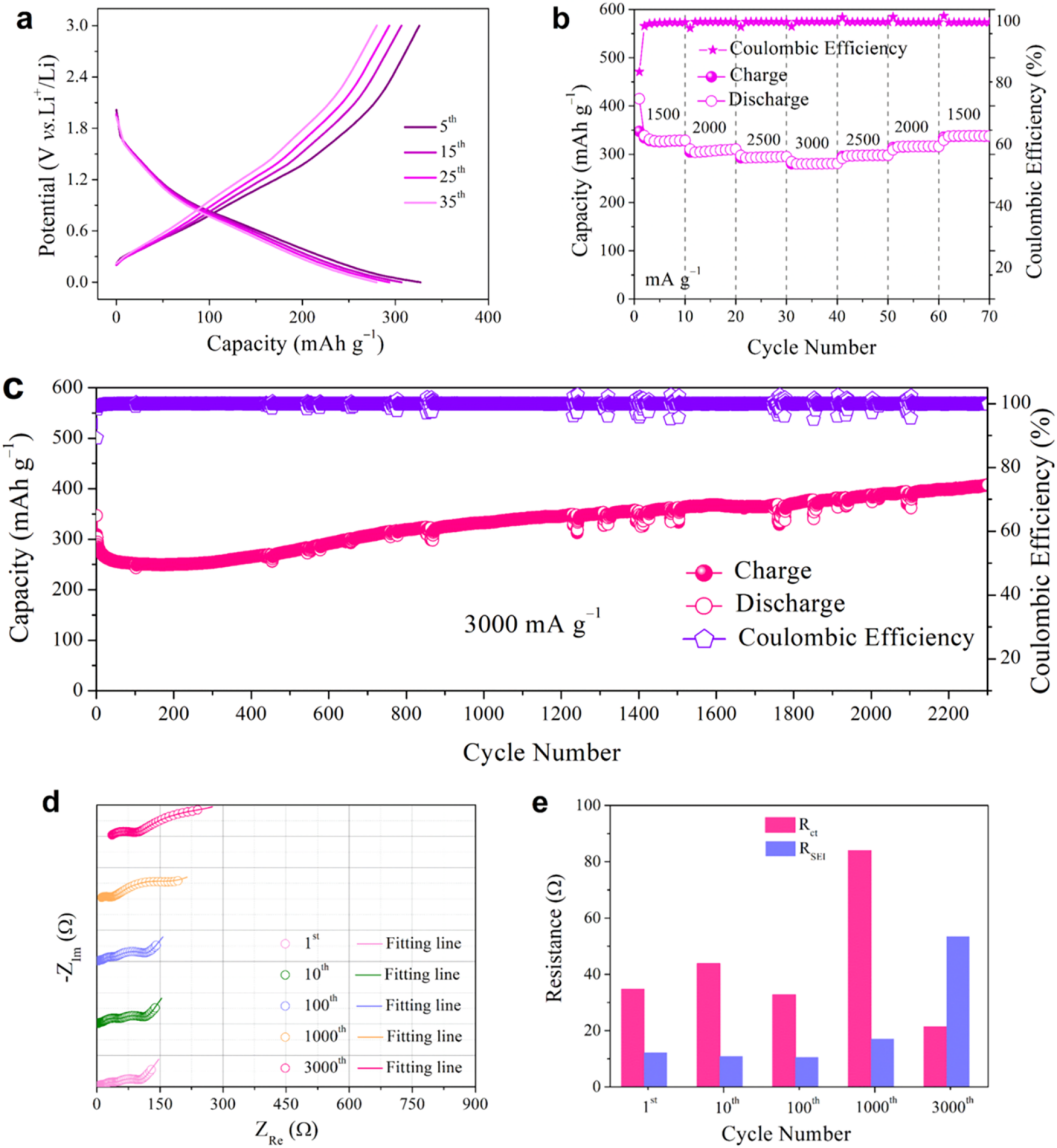
Electrochemical evaluation of the ZrS_3_ electrode in lithium coin cells. (a) Galvanostatic charge/discharge voltage profiles at current densities of 1500, 2000, 2500, and 3000 mA g^–1^. (b) Rate capacity and Coulombic efficiency *vs* cycle number. (c) Long-term cycling performance at 3000 mA g^–1^. (d) EIS spectra recorded at the 1st, 10th, 100th, 1000th, and 3000th cycles. (e) Evolution of *R*_SEI_ and *R*_ct_ derived from EIS fitting at selected cycles.

The ZrS_3_ electrode exhibits outstanding cycling stability even under high current density of 3000 mA g^–1^, as demonstrated in Figure 6c. The initial capacity of 347 mAh g^–1^ gradually declines to 265 mAh g^–1^ by the 400th cycle, primarily due to SEI formation and initial lithium consumption.^51–53^ Subsequently, the capacity slowly recovered and stabilized around 362 mAh g^–1^ by the 1500th cycle, suggesting progressive interfacial stabilization. A continuous increase in capacity was observed during prolonged cycling, reaching 408 mAh g^–1^ at the 2300th cycle, a behavior seldom reported under such high-rate conditions. This can be ascribed to electrode activation, enhanced ionic/electronic conductivity, and progressive interfacial reconfiguration. This is consistent with the “activation” phenomenon reported for sulfide-based electrodes, typically associated with phase transitions and defect-induced increases in conductivity and active reaction sites.^54,55^

The EIS spectra at 3000 mA g^–1^ (Figure 6d) support this observation. As shown in Figure 6e, the fitted *R*_SEI_ and *R*_ct_ values exhibit a dynamic yet well-coordinated evolution. *R*_SEI_ remained relatively stable during the first 100 cycles (10–12 Ω), followed by a moderate increase to ∼17 Ω at the 1000th cycle, and a further rise to ∼53 Ω at the 3000th cycle, suggesting progressive SEI accumulation. In contrast, *R*_ct_ initially fluctuated between 32–44 Ω, then rose to 84 Ω at 1000 cycles, but decreased again to ∼21 Ω at 3000 cycles, indicating interfacial activation and re-established charge-transfer pathways at extended cycling. Such a transient increase followed by a reduction in *R*_ct_ is not uncommon for conversion-type or multiphase electrodes, consistent with the EIS evolution trends reported in previous studies.^56^ These impedance trends reinforce the hypothesis of long-term interfacial reorganization, leading to enhanced capacity retention. The corresponding impedance fitting parameters are summarized in Table 5 of the Supporting Information. These results suggest that ZrS_3_ effectively balances Li^+^ transport and structural durability, demonstrating excellent long-term cycling performance at high current densities.

To understand the structural integrity and surface evolution of the ZrS_3_ electrode, *ex-situ* SEM, SEM-EDX, XPS, and cross-sectional SEM analyses were conducted after 3000 cycles under 3000 mA g^–1^ (Figure S4–S5). XPS spectra (Figure S4) provide additional insights, showing a slight shift in the Zr 3d peak, suggesting limited surface ZrO_2_ formation. This oxide layer is confined to the surface and is not expected to act as an active Li-storage phase but may contribute to interfacial stabilization during prolonged cycling by suppressing continuous side reactions. These results suggest that despite interfacial changes, the electrode retains its structural framework, contributing to stable performance. The SEM micrographs (Figure S5a) reveal that, despite minor surface roughening and moderate aggregation, the electrode remains mechanically stable, with no significant cracks. SEM-EDX analysis (Figure S5b) indicates a gradual increase in oxygen content and a corresponding decrease in sulfur intensity, suggesting mild oxidation of the ZrS_3_ surface. Cross-sectional SEM images (Figure S5c) further confirm that the electrode maintains a well-retained active material layer, which is conducive to sustained ion-transport pathways and high electrochemical reversibility, rather than indicating structural degradation. A notable capacity decay was observed at 60 °C (Figure S8), confirming the thermal instability of ZrS_3_ during cycling. This degradation is consistent with the thermal instability of LiPF_6_-based SEI, in which elevated temperature accelerates electrolyte decomposition and interfacial side reactions, leading to SEI reconstruction, increased impedance, and capacity fading.

To further elucidate the kinetic characteristics of the ZrS_3_ electrode, cyclic voltammetry (CV) measurements were systematically performed over a range of scan rates from 0.4 to 20 mV s^–1^, as illustrated in Figure 7a. In electrochemical systems, charge storage generally consists of two components: faradaic (diffusion-controlled) processes, which involve charge transfer reactions, and nonfaradaic (surface-controlled) contributions, such as double-layer capacitance. These components can be analyzed by evaluating redox peak currents (*i*) at varying scan rates (*v*) using the following relationships^57,58^

3 $$i\left(V\right)=av^{b}$$

4 log⁡i(V)=log⁡a+blog⁡v

**Figure 7:**
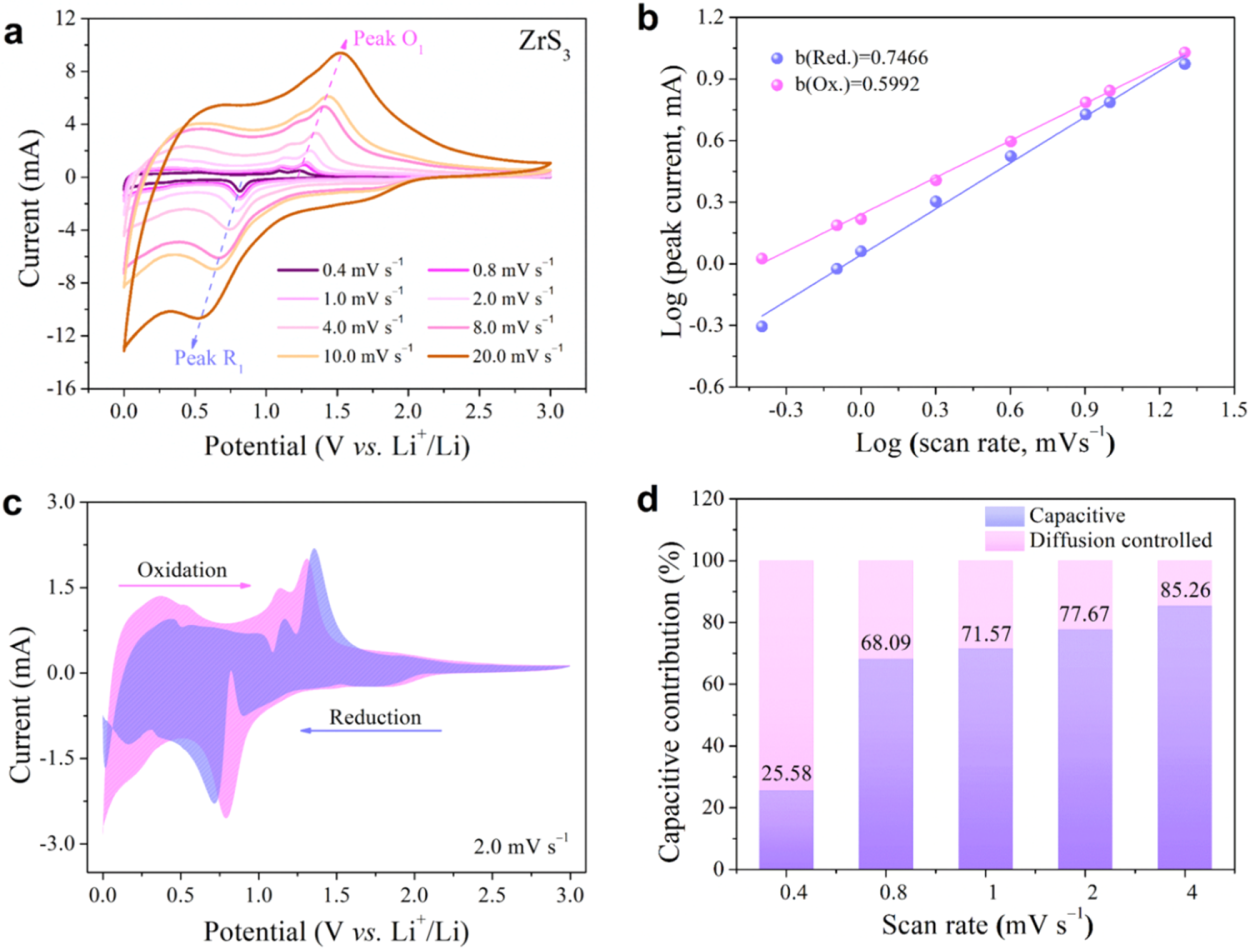
Electrochemical kinetics of the ZrS_3_ electrode. (a) CV profiles at various scan rates. (b) Linear relationship between log (i) and log (v) for each redox peak. (c) Capacitive and diffusion-controlled contributions at a scan rate of 2.0 mV s^–1^. (d) Normalized capacitive contribution and the diffusion-controlled contribution fractions across various scan rates.

In these equations, *a* and *b* are constants where the value of *b* provides insight into the charge storage mechanism.^57–59^ A *b* value close to 1 suggests surface-controlled, capacitive behavior, while a value around 0.5 indicates a diffusion-limited process.

The plots of log* i* versus log* v* in Figure 7b reveal slopes, or “*b*” values, of approximately 0.7466 for the reduction peak (R) and 0.5992 for the oxidation peak (O). These values suggest that the charge storage in ZrS_3_ involves a combination of both capacitive (surface-related) and diffusion-limited behaviors, with capacitive processes making a major contribution. Specifically, the *b* value for the anodic peak 0.7466 highlights a hybrid storage mechanism: capacitive contributions likely originate from accessible active sites associated with the layered morphology and intrinsic defects of ZrS_3_, which may facilitate Li-ion interaction at the surface or edge regions, while the bulk of the material supports diffusion-controlled lithium storage. This dual mechanism of charge storage indicates efficient Li^+^ kinetics and rapid charge transfer near the electrode surface, contributing to the enhanced electrochemical performance and stability of the ZrS_3_ electrode in LIBs applications.

The coexisting capacitance-controlled and diffusion-controlled processes in the ZrS_3_ electrode are characteristic of complex electrochemical reactions, each contributing uniquely to overall lithium storage. These contributions can be quantitatively assessed using the following relationship^57,60^

5 $$i\left(V\right)=k_{1}v+k_{2}v^{0.5}$$

In this equation, the terms *k*_1_ *v* and *k*_2_ *v* ^0.5^ represent the capacitance-controlled (surface-driven) and diffusion-controlled (bulk-driven) contributions, respectively. The parameters *k*_1_ and *k*_2_ are determined by rearranging the eq 5 to analyze the plot of *i*(*V*)/*v* ^0.5^ versus *v* ^0.5^.^57,61,62^

6 $$\frac{i\left(V\right)}{v^{0.5}}=k_{1}v^{0.5}+k_{2}$$

Here, the slope of this plot represents *k*_1_, indicating the extent of surface-controlled behavior, while the y-intercept corresponds to *k*_2_, revealing the diffusion-controlled contribution. By selecting a scan rate of 2.0 mV s^–1^, the capacitance-controlled contribution for the ZrS_3_ electrode was calculated to be 77.67%, as illustrated in Figure 7c. This substantial capacitance suggests that lithium-ion storage in ZrS_3_ is significantly influenced by pseudocapacitive contributions, primarily governed by fast intercalation-related kinetics and surface or near-surface redox processes rather than conventional high-surface-area-driven capacitive storage, which can enable both rapid and stable Li^+^ uptake, especially at higher current densities.^58,62^

Figure 7d reveals the distribution of capacitive and diffusion-controlled contributions over a range of scan rates from 0.4 to 20.0 mV s^–1^. With increasing scan rate, the contribution from capacitance-controlled processes progressively rises from 25.58% at the lowest scan rate to 89% at the highest, highlighting the strong rate capability of the ZrS_3_ electrode.^58,61^ This trend highlights the 2D layered structure of the electrode, which facilitates rapid electron transfer and Li^+^ diffusion by providing accessible active sites at surfaces and edges, together with shortened ion transport pathways.^62^

The diffusion pathways and energy barriers of Li^+^ in bulk and monolayer ZrS_3_ were calculated using the CI-NEB method, as shown in Figure 8a–c. The results indicate that Li^+^ migrates between two adjacent stable H-sites *via* intermediate T-sites, overcoming energy barriers of 0.12 eV in bulk and 0.33 eV in monolayer ZrS_3_, respectively. This suggests that bulk ZrS_3_ possesses intrinsically more favorable Li^+^ migration kinetics, which may contribute to its superior charge–discharge rate performance observed experimentally. Notably, this trend is contrary to many conventional layered materials where monolayers typically facilitate faster ion transport.^63,64^ The monolayer result is included as a theoretical reference to highlight the unusual bulk-favored diffusion in ZrS_3_, rather than as a direct model of the tested electrode. It should be emphasized that these DFT calculations are based on an ideal, pristine ZrS_3_ lattice to probe intrinsic Li^+^ transport. In practice, the electrode surface may undergo oxidation (*e.g.*, ZrO_2_ formation), sulfur loss (as observed in Figure 1e), and SEI formation during cycling; such interfacial and compositional changes can locally influence Li^+^ migration barriers, particularly near the surface. These effects are not captured in the present model and will be assessed in future work.

**Figure 8:**
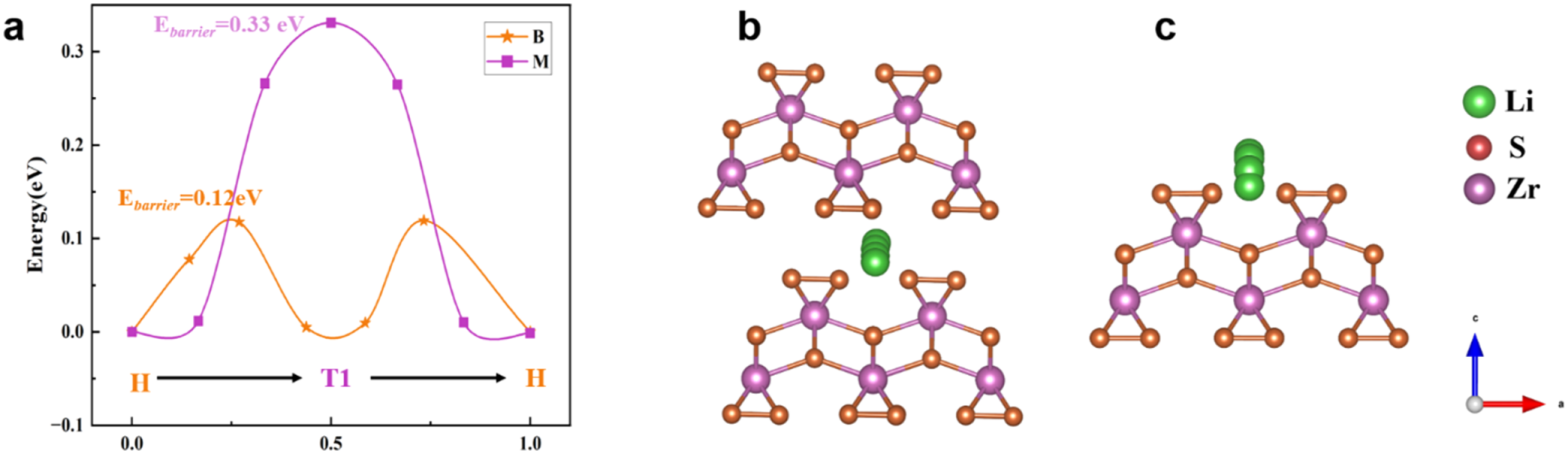
(a) Li-ion diffusion energy barriers in bulk and monolayer ZrS_3_. (b) Diffusion pathway in bulk ZrS_3_. (c) Diffusion pathway in monolayer ZrS_3_.

## 3 Conclusion

2D ZrS_3_ microbelts with a quasi-1D structure were synthesized *via* a straightforward solid-state reaction and evaluated as a promising anode material for lithium-ion batteries. It was found that the ZrS_3_ electrode exhibits a reversible intercalation process at higher potentials, while conversion reactions are activated at lower voltages, with partial structural reversibility upon charging. The ZrS_3_ electrode exhibits excellent lithium storage performance, including a high reversible capacity, superior rate capability (a stable discharge capacity of 281 mAh g^–1^ at a high current density of 3000 mA g^–1^), and outstanding long-term cycling stability (408 mAh g^–1^ at 3000 mA g^–1^ over 2300 cycles). This superior performance can be attributed to (1) the isotropic characteristics of the mesoporous products formed during initial lithiation, which stabilize the electrode structure; (2) fast pseudocapacitive redox processes, which enhance charge-storage kinetics; and (3) low charge transfer resistance, which facilitates efficient electron and ion transport. Overall, this work highlights the potential of quasi-1D ZrS_3_ as a high-performance anode material for lithium-ion batteries, particularly in applications requiring high-rate capability and long-term cycling stability. More broadly, the insights into voltage-dependent intercalation–conversion behavior and bulk-favored ion-transport kinetics provide useful guidance for the rational design of advanced transition-metal chalcogenide anodes.

## 4 Experimental Section

### 4.1 Chemicals

Carbon black (CB, Super P, Alfa Aesar), poly­(vinylidene fluoride) (PVDF, Sigma-Aldrich), *N*-methylpyrrolidone (NMP, 99.7%, Sigma-Aldrich), and ethanol (C_2_H_5_OH, 99.8%, Sigma-Aldrich) were of analytical grade and directly used. The 1.0 M lithium hexafluorophosphate (LiPF_6_) solution mixed with ethylene carbonate (EC), dimethyl carbonate (DMC), diethyl carbonate (DEC) (V/V/V = 1:1:1) with 1.0% vinylene carbonate (VC) was procured from Sigma-Aldrich.

### 4.2 Synthesis of Zirconium Trisulfide (ZrS_3_)

ZrS_3_ crystals were synthesized through a direct reaction between zirconium (Zr) and sulfur (S) in quartz ampoule. In a typical procedure, zirconium (-100 mesh, 99.9%, Alfa Aesar, Germany) and sulfur (99.9999%, 2–6 mm, Wuhan Xinrong New Materials Co., China) were mixed in stochiometric ratio corresponding to 20 g of ZrS_3_ together with 0.7 g of iodine (99.9%, Fisher Scientific, Germany) and melt sealed under high vacuum by oxygen–hydrogen welding torch in quartz ampoule (40 × 250 mm^2^). The ampoule was first heated in muffle furnace on 500 °C for 25 h and on 600 °C for 50 h and on 700 °C for 50 h. Heating and cooling rate were 1 °C/min. Subsequently the ampoule was placed in two zone furnace. First the growth zone was heated on 800 °C and source zone on 600 °C for 2 days. Subsequently the thermal gradient was reversed and growth zone were heated on 700 °C and source zone on 800 °C for 14 days. Finally, the ampoule was cooled on room temperature and crystals collected in argon glovebox.

### 4.3 Computational Details

The first-principles calculations were performed by using density functional theory (DFT) implemented in the Vienna *ab initio* simulation package (VASP).^65,66^ The exchange–correlation function was described by using the Perdew–Burke–Ernzerhof (PBE) of generalized gradient approximation (GGA).^67^ The energy cutoff point of the plane wave basis was set to 650 eV. A 2*a* × 3*b*× 1*c* supercell with three-dimensional periodic boundary conditions was created for bulk ZrS_3_, and a 2*a* × 3*b* monolayer of ZrS_3_ with 20 Å vacuum along the *c*-direction was created. Each atom in the unit cell was completely relaxed until the force on each atom was less than 0.01 eV/Å^–1^, and the electronic self-consistent convergence was set to 10^–6^ eV. The Γ-centered 3 × 3 × 1 *k*-point grid and 3 × 3 × 4 *k*-point grid were used for 2D sheet and bulk ZrS_3_, respectively. The van der Waals (vdW) interactions were corrected by using the DFT-D3 method in bulk ZrS_3_ lattice optimization.^68^ The elastic constants were calculated using stress–strain relationships and the Brillouin-zone (BZ) sampling was carried out with a 5 × 5 × 6 Γ-centered *k* mesh. The method of climbing-image nudged elastic band (CI-NEB) was applied to examine the diffusion pathways and diffusion barriers.^69^

### 4.4 Material Characterization

X-ray diffraction (XRD) patterns of the ZrS_3_ samples were obtained using Bruker D8 Advance Diffractometer (Germany) with Cu Kα radiation over a 2θ angle range from 5° to 80° with a scanning step size of 0.02°.

The morphology of the as-synthesized ZrS_3_ was analyzed using scanning electron microscopy (SEM) with a field emission gun (FEG) electron source (Tescan LYRA dual-beam microscope) at an accelerating voltage of 10 kV. Energy-dispersive X-ray spectroscopy (EDX) was employed for elemental composition analysis and mapping, using an 80 mm^2^ silicon drift detector (SDD) (Oxford Instruments) and Aztec Energy software at an accelerating voltage of 10 kV. The bulk material was deposited on carbon tape and examined without any modifications.

Changes in electrode thickness during cycling were also examined using a Tescan MAIA dual-beam microscope under identical conditions. For cross-sectional analysis, the electrodes were first immersed in liquid nitrogen for cutting and then transferred to carbon tape for SEM observation. EDX analysis was performed at 15 kV using the same detector and software.

Transmission electron microscopy (TEM) micrographs were acquired using a JEOL JEM-1010 instrument at an accelerating voltage of 200 kV. Images were captured with an SIS MegaView III digital camera (Soft Imaging Systems) and analyzed using AnalySIS v. 2.0 software. A suspension of ZrS_3_ was prepared, and the samples were drop-cast onto TEM grids (Cu, 200 mesh, Formvar/carbon, TED PELLA, Inc.) and dried overnight at room temperature.

X-ray photoelectron spectroscopy (XPS) analysis was carried out using an Omicron Nanotechnology Ltd. instrument (Germany) equipped with a monochromatic Al X-ray radiation source (Kα1 = 1486.7 eV). Raman spectroscopy was conducted using a Renishaw inVia system (UK) equipped with a charge-coupled device detector and a 532 nm green DPSS laser, with an output power of 50 mW. Measurements were performed under ambient conditions using a 20× objective lens, with each acquisition set to a 10-s integration time and a laser power of 10 mW. The specific surface area and pore size distribution were measured using a BET analyzer (NOVAtouch 2200, USA) through nitrogen physisorption at 77 K.

### 4.5 Battery Assembly and Electrochemical Measurements

The electrode slurry was prepared by combining the active material (70 wt %), CB conductive additive (20 wt %), and PVDF binder (10 wt %) in NMP solvent. Subsequently, the homogeneous slurry was coated onto a copper foil current collector using a laboratory doctor blade. The coated slurry was then dried in a heat pad oven at 70 °C for 12 h. The dried electrode was punched into 10 mm diameter wafers for use with the loading mass of 1.5–2.2 mg cm^–2^. Metallic lithium (Li) was employed as the counter electrode. Glass microfiber film (Whatman, grade GF/D) was used as the separator. The electrolyte was 1.0 M LiPF_6_ in a mixed solvent (EC/DMC/DEC) with a volume ratio of 1/1/1 (v/v/v) and 1.0% VC. Two-electrode lithium cells were assembled using CR2025 coin cells from MTI Corporation in an argon-filled glovebox (MBRAUN, Germany, oxygen and water contents <0.1 ppm).

Cyclic voltammetry (CV) measurements on the ZrS_3_ electrode were performed at a scan rate of 0.2 mV s^–1^, covering a voltage range from 1.0 to 3.0, 0.3 to 3.0, 0.001 to 3.0 V vs Li^+^/Li. Multiple CV tests were conducted at scanning rates ranging from 0.4 to 20.0 mV s^–1^. Electrochemical impedance spectroscopy (EIS) measurements were performed under various conditions, including open circuit voltage (OCV) and after 10 cycles at different current densities (1500, 2000, 2500, and 3000 mA g^–1^). The measurements were conducted by applying an AC voltage with an amplitude of 10 mV across a frequency range from 100 kHz to 100 mHz. The impedance spectra were obtained and analyzed using Nyquist plots. Data processing and fitting of the impedance spectra were carried out using Nova 2.1 software and ZView 2 software, respectively. CV measurements, coupled with EIS, were performed on the same coin cell. The tests were conducted using an Autolab PGSTAT204 workstation (Eco Chemie, Utrecht, Netherlands), which provided the necessary data for both CV and EIS analysis.

The cycling behavior and rate capability of the ZrS_3_ electrodes were investigated in CR2025 coin cells, operating within a voltage range of 1.0–3.0, 0.3–3.0, 0.001–3.0 V *vs* Li^+^/Li. The cycling test comprised 40 cycles, with each cycle conducted at a current density of 50 mA g^–1^. In addition to the cycling test, rate performance measurements were performed. The ZrS_3_-based cells underwent cycling at a series of current densities: 25, 1500, 2000, 2500, and 3000 mA g^–1^. Following the maximum current density of 3000 mA g^–1^, the current density was gradually decreased in reverse steps down to 1500 mA g^–1^, with each step consisting of 10 cycles to assess the stability and rate capability of the electrode. The electrode was initially activated at 25 mA g^–1^ for 10 cycles to ensure full lithiation and stabilization. Therefore, this initial activation phase was excluded from the presented data. For assessing long-term cycling stability, the cells were subjected to 3000 cycles at a fixed current density of 3000 mA g^–1^. Additionally, separate long-term cycling tests were performed over 600 cycles at a current density of 1500 mA g^–1^, under controlled temperatures of 25 and 60 °C, to further examine the impact of temperature on the cycling durability and stability of the ZrS_3_ electrodes. All galvanostatic charge–discharge tests were performed at room temperature, utilizing a Neware battery test system (BTX 7.6, Shenzhen, China).

### 4.6 Battery Disassembly and *Ex-Situ* Characterization

After specific cycling protocols, each assembled coin cell was disassembled in an argon-filled glovebox to prevent exposure to air. The recovered electrodes were carefully rinsed with DMC solvent to remove residual electrolyte and surface impurities. Subsequently, *ex-situ* XRD and XPS characterization was conducted to analyze the structural changes in the electrodes post-cycling. Morphological changes in ZrS_3_ after cycling were investigated using SEM with a Tescan MAIA dual-beam microscope at 10 kV. The EDX analysis was performed at 15 kV using the same SDD detector and software. To prepare the samples, the cells were opened, and the electrodes were placed on carbon tape for observation without further modifications.

## Supplementary Material



## Data Availability

The data sets generated during and/or analyzed during the study are accessible via the Zenodo repository: 10.5281/zenodo.15389116.

## Notes

ET surface area and pore structure analysis of ZrS_3_; *ex-situ* XRD patterns at different voltage windows; elastic property calculations; SEM and EDX analyses during cycling; XPS spectra at different cycling stages; rate capability tests; electrochemical impedance spectroscopy analysis and equivalent circuit fitting; temperature-dependent cycling performance (PDF)
