# Supplementary material for: Quasi-1D Chain-Based Zirconium Trisulfide as a Low-Potential High-Rate Anode: Structural and Reaction Mechanism Insights
Source: ACS Appl Mater Interfaces. 2026 Apr 16;18(16):23072–85. doi: 10.1021/acsami.5c22469 (PMC13133779; doi:10.1021/acsami.5c22469)
Supplement: Supplementary file 1 [file am5c22469_si_001.pdf]

SUPPORTING INFORMATION

Quasi-1D Chain-Based Zirconium Trisulfide as a Low-Potential High-Rate Anode: Structural and Reaction Mechanism Insights

Shuangying Wei[a*], Min Liu[b], Ruizhi Yu[c], Haoyang Jiang[d], Huaijuan Zhou[e], Heng Li[a], Min Li[a,f], Payal Chauhan[a], Bing Wu[a], Takeshi Matsuo[g], Kseniia Mosina[a], Lukas Dekanovsky[a], Filipa M. Oliveira[a], Jan Luxa[a], Ondrej Jankovsky[a], Jincang Su[d*], Zdeněk Sofer[a*]

[a] *Department of Inorganic Chemistry, University of Chemistry and Technology Prague, Technická 5, Prague 6, 166 28, Czech Republic*

[b] *College of New Energy, Ningbo University of Technology, Ningbo, Zhejiang 315336, China*

[c] *Institute of Micro/Nano Materials and Devices, Ningbo University of Technology, Ningbo, Zhejiang 315211, China*

[d] *school of Materials Science and Engineering，Xiangtan University，Xiangtan 411105, China*

[e] *Advanced Research Institute of Multidisciplinary Sciences, Beijing Institute of Technology, Beijing 100081, China*

[f] *School of Physics, Xi'an Jiaotong University, Xi'an 710049, China*

[g] *Department of Mechanical Engineering, Okayama University, 3-chōme-1 Tsushimanaka, Kita Ward, Okayama 700-8530, Japan*

Corresponding authors: weis@vscht.cz; sujc@xtu.edu.cn; zdenek.sofer@vscht.cz

Table 1. BET surface area, total pore volume, and average pore size of ZrS_3.

Material	BET surface area ($m^2\ g^{-1}$)	BJH pore volume ($cm^3\ g^{-1}$)	Mean pore size (nm)
ZrS_3	3.505	0.0065	4.038

To investigate the structural evolution and electrochemical reaction mechanisms, *ex-situ* XRD measurements were performed at different voltage windows: 1.0–3.0 V, 0.3–3.0 V, and 0.001–3.0 V (**Figure S1a** to **d**, including full range and selected regions). The observed peak shifts, peak vanishing, and emergence of new peaks provide insights into the lattice stability and possible phase transformations occurring during discharge and charge processes. As shown in the full-range patterns (**Figure S1**a), pristine ZrS_3 exhibits sharp diffraction peaks, indicating high crystallinity. Upon discharge to 1.0 V, peak positions shift slightly, suggesting lattice expansion due to Li-ion intercalation. More pronounced shifts and intensity changes are observed after deeper discharges to 0.3 V and 0.001 V.

In the magnified range of 13–16° (**Figure S1**b), the pristine ZrS_3 electrode exhibits a sharp diffraction peak at 14.8°, indicative of its high crystallinity. After discharging to 1.0 V, this peak disappears, suggesting significant structural distortion or amorphization induced by lithium insertion. Upon subsequent charging to 3.0 V, a broad and weaker peak emerges around 13.9°, implying partial structural recovery but incomplete phase reversibility. When discharged to deeper voltages (0.3 V and 0.001 V), no distinct peaks are observed in the 13–16° range, further indicating severe structural disruption. Notably, after discharge to 0.001 V, two broad features appear around 13.7° and 15.0°, corresponding to the formation of new disordered phases. Upon charging from

0.001 V to 3.0 V, a broadened peak is recovered at approximately 14.1°, suggesting partial reorganization of the lattice but with significant amorphization remaining.

In the 33–36° region (**Figure S1**c), the pristine ZrS_3 electrode exhibits a sharp diffraction peak at 35.1°, characteristic of its highly ordered crystal structure. After discharging to 1.0 V, a new peak appears at approximately 34.2°, suggesting lattice expansion and partial structural distortion. Upon charging to 3.0 V, the peak slightly shifts to around 34.3°, indicating partial lattice recovery. When discharged to 0.3 V, a weak and broadened peak is observed near 33.9°, and after recharging to 3.0 V, no distinct diffraction feature is detected, implying significant structural degradation. Notably, after deep discharge to 0.001 V and subsequent charging, broad peaks persist around 34.2°, further evidencing severe amorphization and incomplete structural reversibility.

In the high-angle region of 60–63° (**Figure S1**d), the pristine ZrS_3 electrode exhibits two split diffraction peaks at 62.2° and 62.3°, characteristic of its highly ordered crystal structure. After discharging to 1.0 V, these peaks disappear, indicating severe structural disruption. Upon charging to 3.0 V, a broad and weakened peak emerges at approximately 61.0°, suggesting partial but incomplete recovery. Further discharge to 0.3 V results in a weak, broadened feature near 60.7°, while no distinct diffraction signal is detected after recharging to 3.0 V. Notably, after deep discharge to 0.001 V, a broad peak reappears near 62.3°, indicating partial retention or reformation of the original phase. However, subsequent charging leads to the disappearance of this feature, suggesting irreversible structural degradation at deep discharge levels.

The *ex-situ* XRD patterns of the ZrS_3 electrode exhibit relatively stable crystalline features, especially within the 1.0–3.0 V window, while partial irreversible structural changes occur at deeper discharge states (0.3 V and 0.001 V). Overall, the *ex-situ* XRD patterns reveal that ZrS_3 undergoes voltage-dependent structural evolution. Moderate discharge (to 1.0 V) mainly involves

partial reversible intercalation, while deeper discharge (to 0.3 V and 0.001 V) induces partial irreversible conversion and amorphization. Despite these transformations, the long-range crystalline framework of ZrS_3 remains partially preserved, supporting its stable cycling performance.

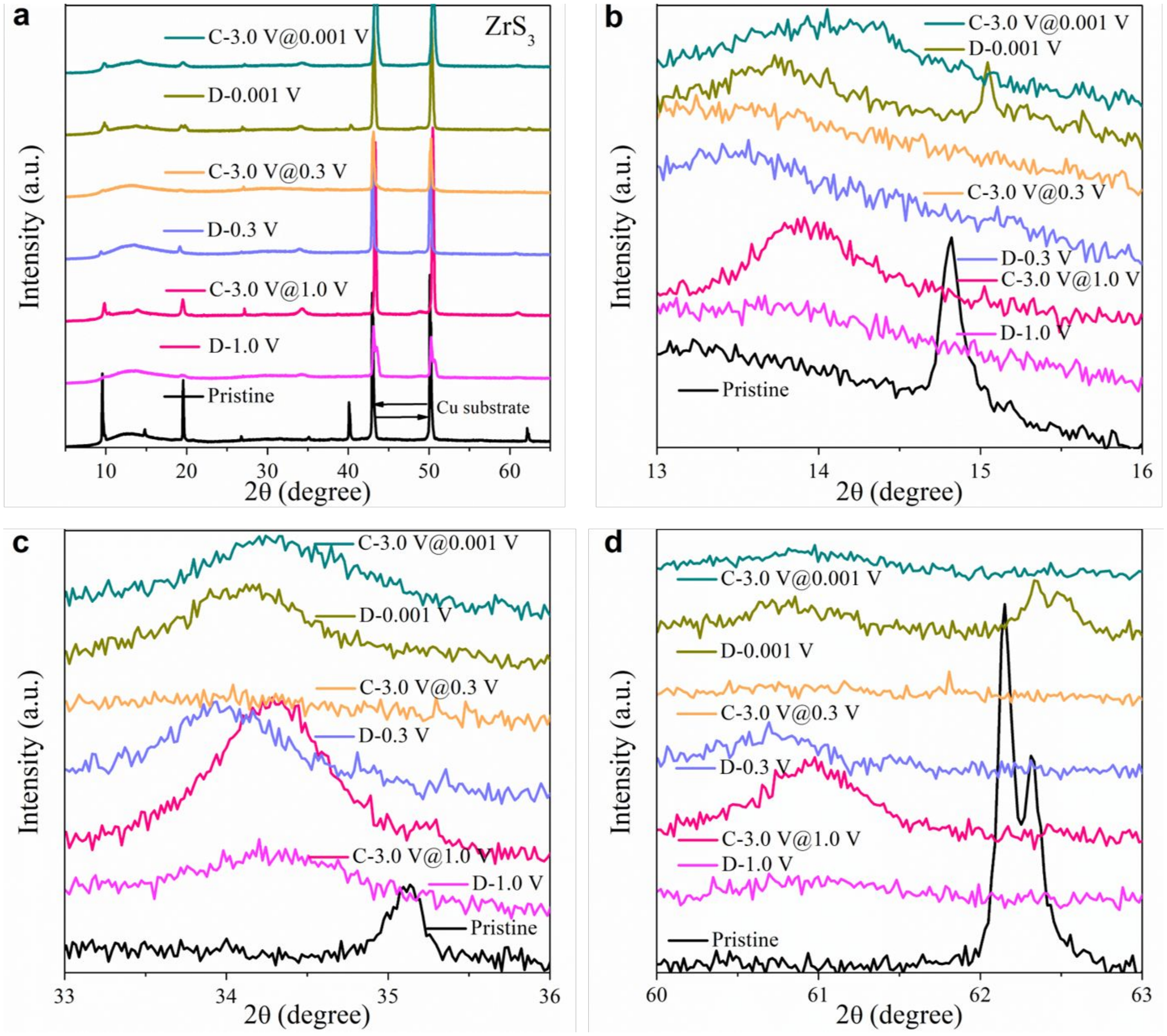

Figure S1. *Ex-situ* XRD analysis of the ZrS_3 electrode after discharge and charge across different voltage windows. (a) Full range (1.0–3.0 V, 0.3–3.0 V, and 0.001–3.0 V), (b) magnified 13–16°, (c) magnified 33–36° and (d) magnified 60–63°.

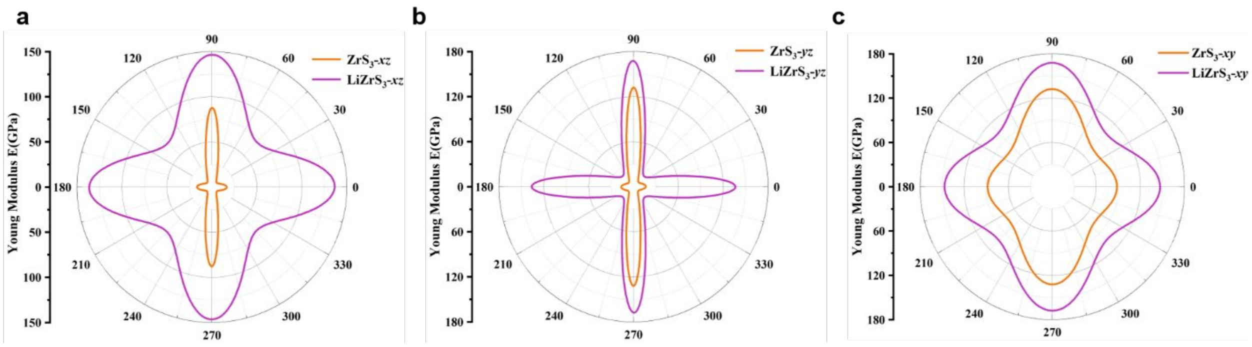

Figure S2. Polar diagrams of Young's modulus for bulk ZrS_3 and lithiated $LiZrS_3$ at different directions (a) *xz*, (b) *yz*, (c) *xy*.

Compared with TiS_3 and NbS_3, ZrS_3 shows the widest ion channels, the weakest interlayer coupling, and the highest air stability, along with a lower discharge potential. These features, combined with superior rate capability and long-term cycling stability, make ZrS_3 a promising anode material for high-energy-density lithium-ion batteries. A comparison of key structural and electrochemical properties of TiS_3, NbS_3 and ZrS_3 is summarized in **Table 2**.

Table 2. Key structural and electrochemical characteristics of TiS_3, NbS_3, and ZrS_3.

Parameter	TiS_3	NbS_3	ZrS_3
Crystal system[1]	Monoclinic	Monoclinic	Monoclinic
M–S bond length (Å)	2.46–2.67[2]	–	2.61–2.72[3]
Interchain spacing (Å)	~4.95	2.619–2.635	~3.06
Band gap (eV)[4]	1.08	1.18	1.92
Air stability	Medium	Low[5]	High
Average discharge potential (V *vs* Li^+/Li)	~1.1–1.5[6]	–	~0.9–1.0 (this work)

SEM images at lower magnifications (**Figures S3**a–d) complement the high-resolution views in the main text by providing an overview of the large-scale morphological evolution of the ZrS_3 electrode during cycling. The pristine electrode (**Figure S3**a) exhibits a densely packed, layered architecture with well-defined flake alignment. After 1 and 10 cycles (**Figures S3**b and **S3**c), the surface becomes noticeably fragmented, with more exposed edges and increased porosity, likely induced by initial lithiation reactions and SEI formation. After 100 cycles (**Figure S3**d), the morphology becomes increasingly disordered, and numerous fine aggregates appear, indicating surface roughening and accumulation of cycling-induced byproducts. These observations confirm the progressive structural evolution and are consistent with the high-magnification images shown in **Figure 4**. SEM-EDX mapping (**Figures S3**e–h) indicates a homogeneous distribution of Zr and S across the electrode surface. Notably, the elemental distribution remains spatially uniform throughout cycling, without clear evidence of phase separation or localized degradation. After 1 and 10 cycles, a slight increase in the Zr signal is observed, while the S signal remains stable, resulting in a slightly decreased Zr/S ratio (from 0.42 to ~0.35), as shown in **Table 3**. This may reflect minor sulfur loss due to initial side reactions, such as electrolyte decomposition or formation of soluble sulfur species. Meanwhile, the O content increases steadily, especially after 100 cycles (O/Zr = 7.11), suggesting accumulation of oxygen-containing surface species, such as ZrO_2 and/or SEI components. Nevertheless, the Zr/S ratio stays within a reasonable range, suggesting good overall material stability during this period, as further confirmed by the quantitative elemental ratios listed in **Table 3**.

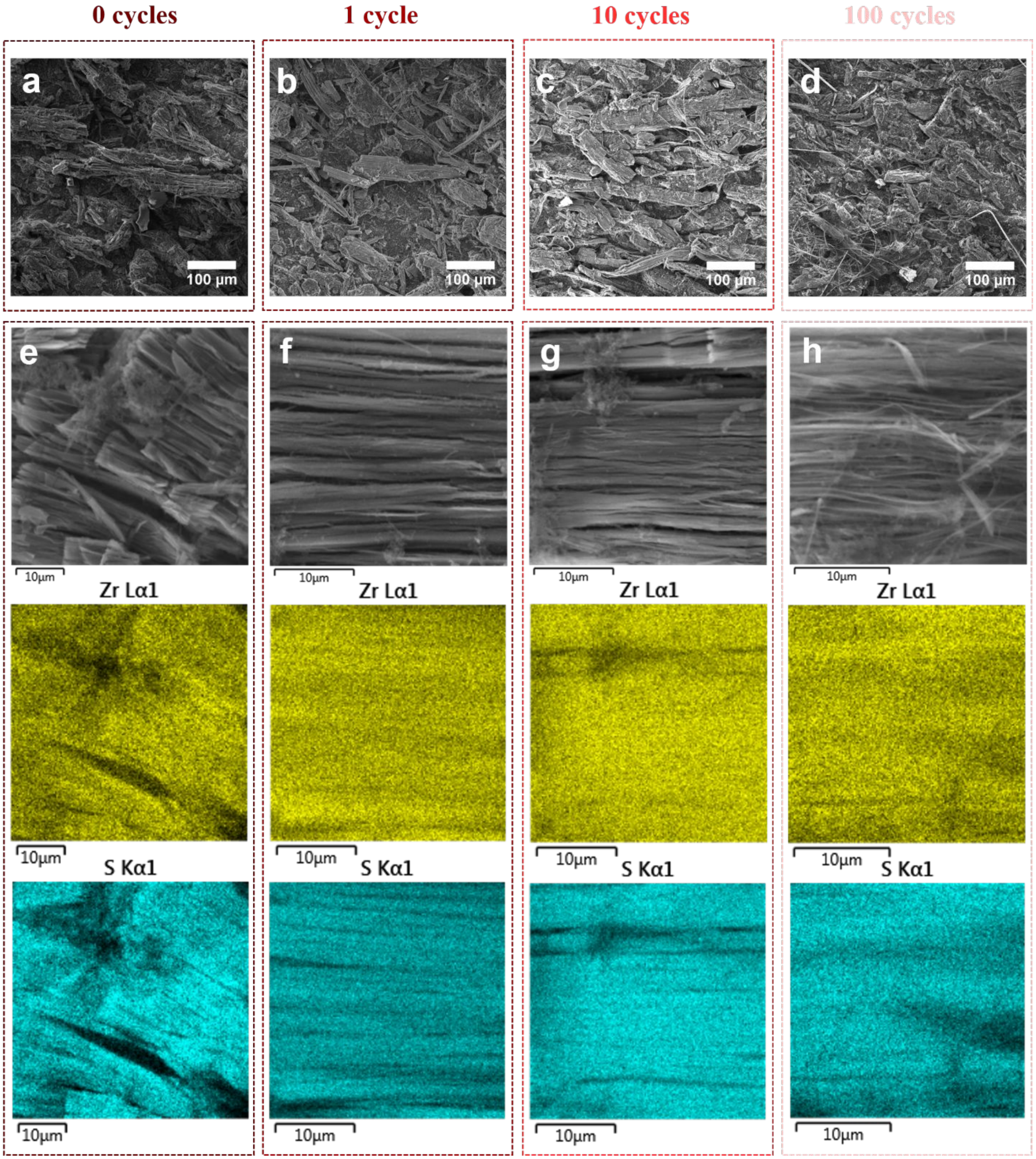

Figure S3. Morphological evolution of ZrS_3 electrodes during cycling at 100 mA g^{-1} (0.001–3.0 V). (a–d) SEM images at low magnifications after 0, 1, 10, and 100 cycles. (e–h) SEM images and corresponding EDX elemental mappings of Zr and S after 0, 1, 10, and 100 cycles.

Table 3. Atomic ratios of Zr, S, and O in ZrS_3 electrodes at different cycle numbers.

Cycle number	Zr (At%)	S (At%)	O (At%)	Zr/S Ratio	O/Zr Ratio
0	13.8%	33.2%	53%	0.42	3.84
1	15.7%	42.4%	41.8%	0.37	2.66
10	15.6%	44.2%	40.2%	0.35	2.58
100	9.5%	23%	67.5%	0.41	7.11
3000	5.8%	12.5%	81.4%	0.46	14.0

To gain insights into the interfacial chemical evolution of ZrS_3 during cycling, XPS analysis was performed on electrodes at various cycling stages under different current densities (**Figure S4**). The Zr 3d spectra of the pristine electrode display two sharp peaks at 181.9 and 184.3 eV, corresponding to Zr^{4+} in ZrS_3. After the first cycle, the Zr 3d peaks slightly broaden and exhibit weak shoulder features at lower binding energies, indicative of partial surface reduction and structural reconstruction.[7] As cycling progresses, particularly after 100 and 3000 cycles, the Zr 3d signal intensity gradually declines, and the peaks become less defined, suggesting increased disorder or surface coverage by SEI components. The O 1s spectra show a gradual intensity increase after cycling, with the appearance of a shoulder at ~532.8 eV, which can be assigned to organic SEI species such as $ROCO_2Li$ and Li_2CO_3 resulting from electrolyte decomposition.[8] These features become more prominent with prolonged cycling, consistent with the continuous accumulation of interfacial byproducts. The F 1s signal is absent in the pristine electrode but emerges after the first cycle, centered on ~685.6 eV, characteristic of LiF.[9] The signal also reaches its maximum after the first cycle and gradually decreases with extended cycling, indicating the

initial formation and subsequent stabilization or consumption of LiF in the SEI layer. The Li 1s spectra follow a similar trend: a moderate increase in intensity after the first cycle, corresponding to the accumulation of lithium-containing SEI components (*e.g.*, LiF, Li_2CO_3, LiOH),[10] followed by a decrease after long-term cycling, suggesting redistribution or partial dissolution of lithium species. It should be noted that S 2p spectra were not acquired in this work, so direct detection of the Li_2S characteristic peak at ~161.2 eV is unavailable; nevertheless, the combined Zr 3d, O 1s, F 1s, and Li 1s evolution provides indirect evidence of interfacial reactions consistent with partial conversion to sulfide species. These results collectively confirm the progressive evolution of the electrode–electrolyte interface during cycling. Under moderate current densities (*e.g.*, 100 mA g^{-1}), the ZrS_3 electrode preserves good structural integrity. In contrast, at high rates (*e.g.*, 3000 mA g^{-1}), substantial interfacial modification occurs, including SEI thickening and redox-induced surface alteration, which may influence long-term electrochemical stability.[11]

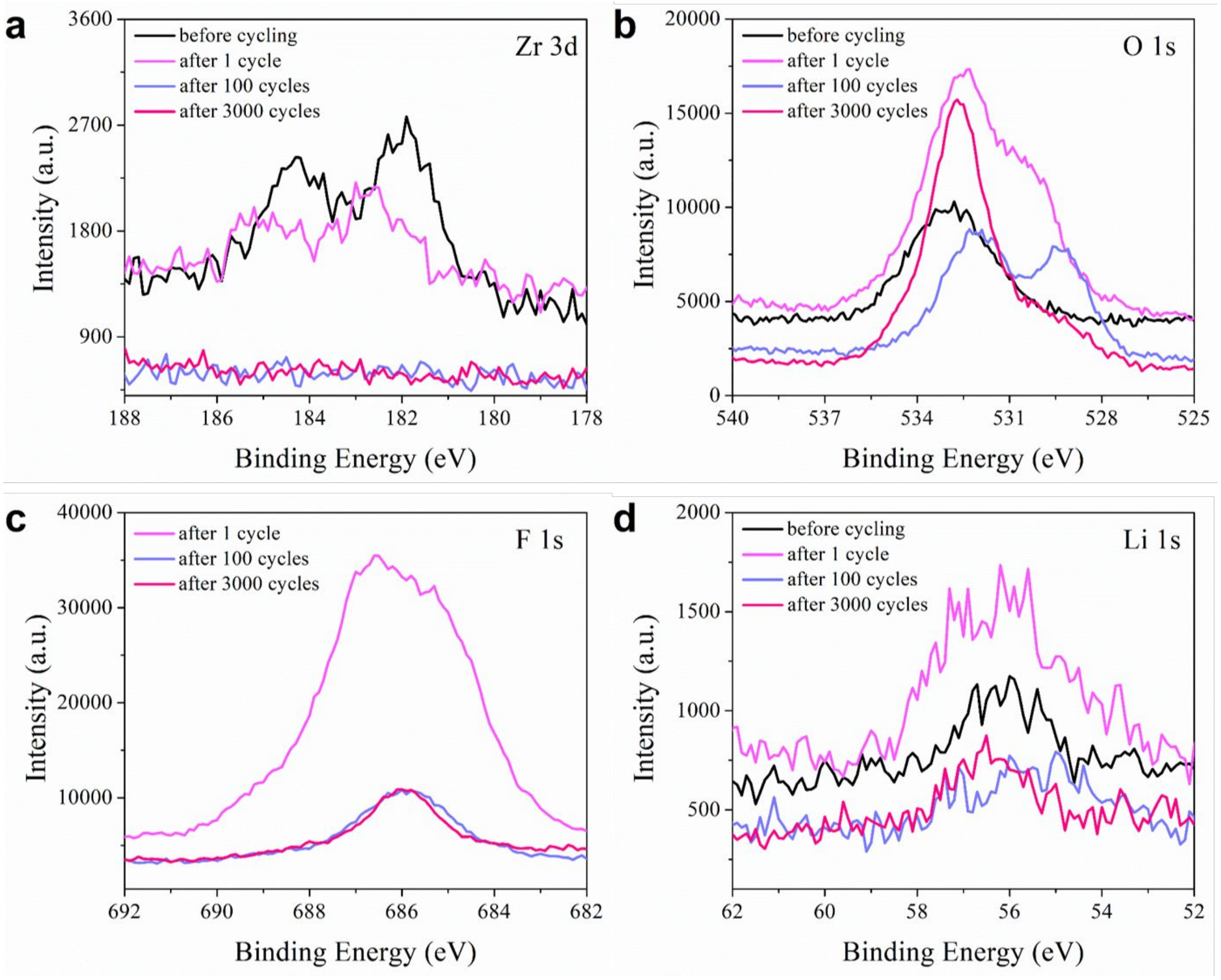

Figure S4. XPS spectra of the ZrS_3 electrodes at different cycling stages. (a) Zr 3d, (b) O 1s, (c) F 1s, and (d) Li 1s spectra collected before cycling, after 1 cycle and 100 cycles at 100 mA g^{-1} and 3000 cycles at 3000 mA g^{-1}.

To assess the rate capability of the ZrS_3 electrode, galvanostatic charge/discharge tests were conducted at progressively increasing current densities ranging from 25 to 3000 mA g^{-1}. The ZrS_3 electrode exhibited stable voltage profiles with gradually decreasing capacities, reflecting expected kinetic constraints under high-rate conditions (**Figure S6**a). The electrode delivered discharge capacities of approximately 886, 847, 654, 507, 413, 349, 320, and 267 mAh g^{-1} at 25, 50, 100,

250, 500, 1000, 1500, and 3000 mA g^{-1}, respectively (**Figure S6**b). As expected, the capacity decreased with increasing current due to increasing kinetic limitations. Such performance at 3000 mA g^{-1}, coupled with stable voltage profiles, highlights the outstanding rate tolerance of the ZrS_3 electrode. The Coulombic efficiency increased rapidly after the initial activation process, reaching >95% within 10 cycles. During the stepwise increase in current density, a transient drop in CE was observed in the first cycle after each current change (*e.g.*, 89.5% at 250 mA g^{-1}), attributed to temporary interfacial reconfiguration and kinetic adaptation. In subsequent cycles, CE consistently stabilized above 98%, with most values exceeding 99.5%, confirming the high electrochemical reversibility.

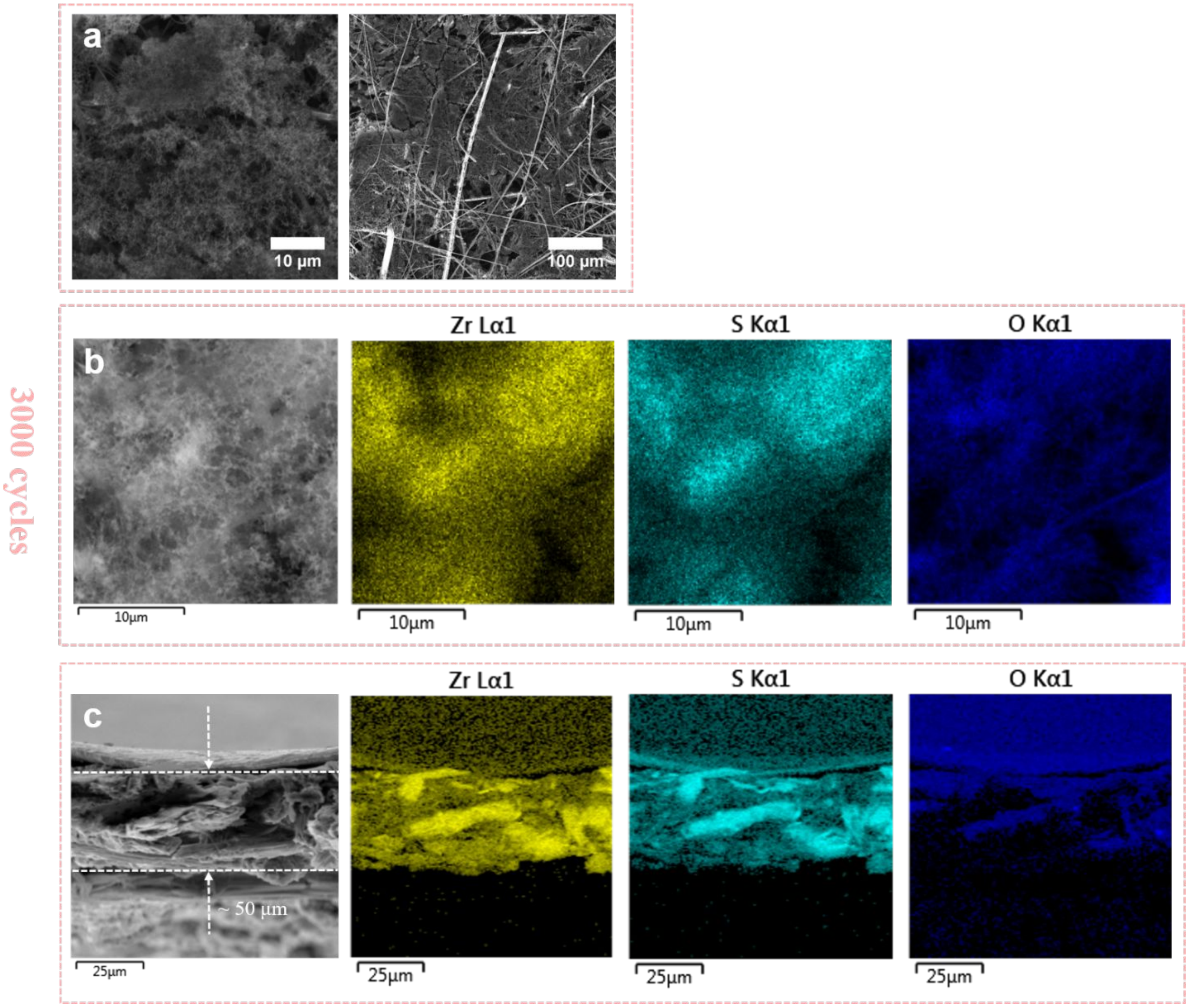

Figure S5. (a) Top-view SEM image, (b) SEM images with corresponding EDX elemental mappings, and (c) cross-sectional SEM images with corresponding EDX elemental mappings of the ZrS_3 electrode after 3000 cycles at 3000 mA g^{-1}.

Note: This set of rate tests was performed without a final recovery step to low current density; thus, the results represent the irreversible rate capability under continuous high-rate cycling.

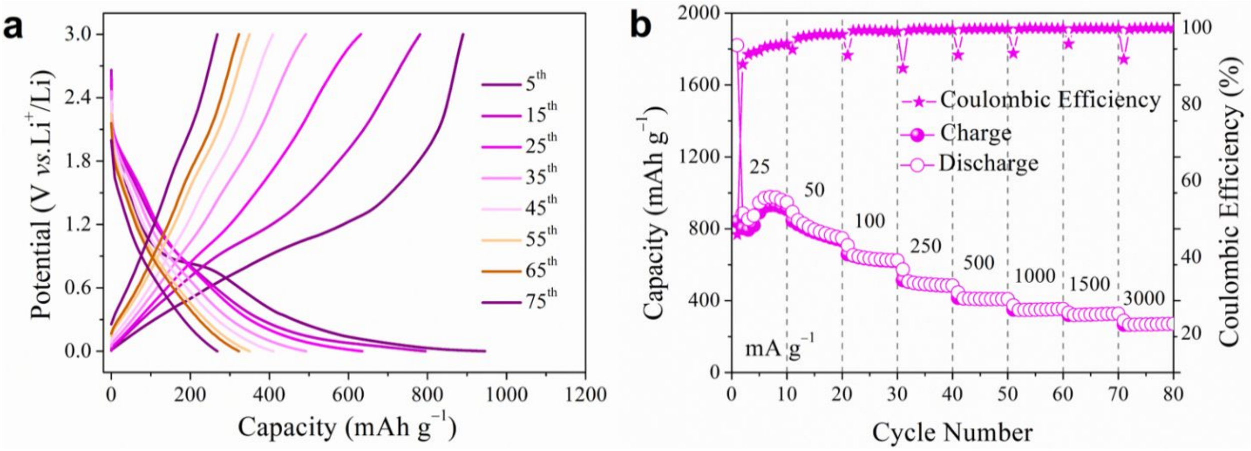

Figure S6. Rate capability of the ZrS_3 electrode tested at various current densities ranging from 25 to 3000 mA g^{-1}. (a) Voltage profiles. (b) Rate performance and Coulombic efficiency.

To gain quantitative insight into the evolution of interfacial properties during cycling, EIS spectra were collected and fitted under various conditions. The pristine electrode (0th cycle, open-circuit) was well described by a simple Randles-type model ($R_s(R_{ct}\|CPE_1)W_s$), showing a large R_{ct} (~597 Ω, **Table S4**), consistent with relatively slow charge-transfer kinetics. After the first cycle at 25 mA g^{-1}, an SEI component (R_{SEI}) emerged (~316 Ω), while R_{ct} decreased moderately. Upon further cycling (10 cycles), the system evolved into a more complex three-time-constant circuit with separate R_{SEI}, R_{ct}, and R_4 components. This is interpreted as stable SEI formation and enhanced charge-transfer (R_{ct} = 27 Ω), and an additional interfacial process likely related to film growth or phase reorganization (R_4 ≈148 Ω).

The SEM and cross-sectional images (**Figures 4**a–l) at corresponding stages reveal a progressive development of a uniform surface layer, accompanied by increased oxygen signal in the O Kα mapping (**Figures 4**e–h), indicative of electrolyte decomposition products. XPS analysis (**Figure**

S4) further confirms the accumulation of LiF (F 1s, 685 eV) and organic lithium compounds (O 1s, ~531 eV) after 1–100 cycles, which are known to contribute to the observed R_{SEI} component in EIS.

At higher current densities (1500–3000 mA g^{-1}), the same circuit topology was maintained, but both R_{SEI} and R_{ct} progressively decreased (R_{SEI} = 11–14 Ω, R_{ct} = 33–45 Ω), suggesting improved ion accessibility and reduced interfacial polarization. The Warburg impedance (W_s), reflecting ion diffusion within the electrode bulk, remained relatively unchanged across these high current densities, as indicated by the parallel low-frequency slopes in the Nyquist plots (**Figure S7**b), confirming that Li^+ transport pathways within the ZrS_3 electrode were largely preserved even under fast cycling conditions.

After extended cycling at 3000 mA g^{-1}, despite the increase in both R_{SEI} and R_{ct} at the end of 3000 cycles (**Table S**4), the discharge capacity continues to rise (**Figure 6**c), indicating that the higher resistance does not significantly limit overall capacity delivery under high-rate conditions. This can be attributed to morphological and compositional evolution revealed by SEM and XPS: particle fragmentation and porosity increase (**Figures S**4 and 5) expose fresh active surfaces, while the formation of LiF- and Li_2CO_3-rich SEI (**Figures S**4 and 5) may stabilize these new interfaces, enabling more complete utilization of the electrode material.

The overall impedance reduction with cycling and increased current clearly correlates with the observed high-rate capability (**Figure 6**), highlighting fast lithium ion/electron transport and structural adaptability. Furthermore, the observed impedance evolution and additional interfacial resistance ($R_4 \approx$ 52–64 Ω at high rates) suggest interfacial reorganization or phase transitions occurring at elevated current densities. Such interfacial reorganization, when accompanied by structural activation, can lead to simultaneous resistance growth and capacity enhancement, as

previously reported in some conversion-type and layered sulfide electrodes (*e.g.*, MoS_2, FeF_2).[12–14] All fits showed excellent agreement between model and data ($\chi^2 \lesssim 10^{-4}$). The equivalent circuits for different stages are summarized in **Figure S7**c. These results highlight that both SEI stability and reduced charge-transfer resistance contribute synergistically to the excellent rate performance of the ZrS_3 electrode. The post-cycling morphological and chemical analyses therefore substantiate the EIS-derived interpretation, linking resistance changes to SEI growth, composition evolution, and possible structural rearrangements at the electrode–electrolyte interface.

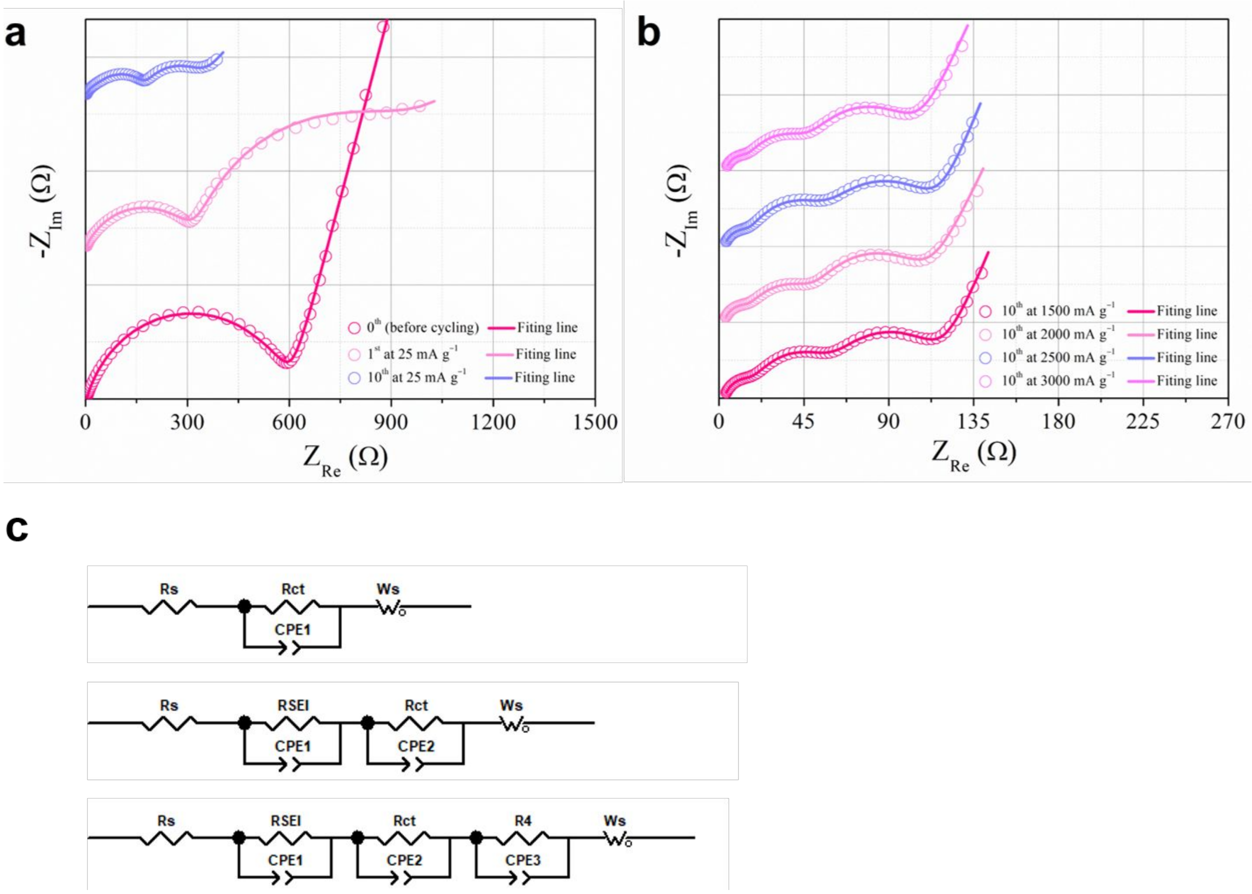

Figure S7. EIS spectra of ZrS_3 electrodes fitted using a modified Randles-type equivalent circuit. (a) EIS spectra recorded at OCV and after the 1st and 10th cycles at 25 mA g^{-1}. (b) Rate-dependent EIS spectra after 10 cycles at 1500–3000 mA g^{-1}. (c) Equivalent circuit models used for fitting: (top) pristine electrode (0th); (middle) cycled electrode at low current (1st at 25 mA g^{-1}); (bottom) cycled electrode after 10 cycles at 25 mA g^{-1}, 1500, 2000, 2500 and 3000 mA g^{-1}.

Table S3. Summary of key impedance parameters from EIS fitting at selected cycles under various current densities. Impedance parameters of the ZrS_3 electrode obtained from EIS fitting using a modified Randles circuit (**Figure S7**c) at different stages, including the initial activation (OCV, 25 mA g^{-1}) and after 10 cycles under various current densities (1500–3000 mA g^{-1}). The table summarizes the fitted values of R_s (solution resistance), R_{SEI} (SEI resistance), R_{ct} (charge-transfer resistance), and R_4 (additional interface resistance), along with the χ^2 fitting accuracy.

**Condition**	**Equivalent circuit**	**R_s (Ω)**	**R_{SEI} (Ω)**	**R_{ct} (Ω)**	**R_4 (Ω)**	**χ^2**
0 (Before cycling)	$R_s(R_{ct}\,CPE_1)W_s$	2.961	/	596.9	/	8.1×10^{-4}
1 cycle (25 mA g^{-1})	$R_s(R_{SEI}CPE_1)(R_{ct}CPE_2)W_s$	1.419	315.7	529.2	/	7.6×10^{-4}
10 cycles (25 mA g^{-1})	$R_s(R_{SEI}CPE_1)(R_{ct}CPE_2)(R_4CPE_3)W_s$	1.525	148.1	26.96	147.9	9.1×10^{-5}
10 cycles (1500 mA g^{-1})	$R_s(R_{SEI}CPE_1)(R_{ct}CPE_2)(R_4CPE_3)W_s$	2.91	14.11	44.67	51.52	9.8×10^{-5}
10 cycles (2000 mA g^{-1})	$R_s(R_{SEI}CPE_1)(R_{ct}CPE_2)(R_4CPE_3)W_s$	2.67	12.5	36.27	63.72	1.3×10^{-4}
10 cycles (2500 mA g^{-1})	$R_s(R_{SEI}CPE_1)(R_{ct}CPE_2)(R_4CPE_3)W_s$	2.87	12.79	42.55	53.85	1.0×10^{-4}
10 cycles (3000 mA g^{-1})	$R_s(R_{SEI}CPE_1)(R_{ct}CPE_2)(R_4CPE_3)W_s$	3.425	11.47	33.66	52.31	1.0×10^{-4}

Table S4. Impedance parameters of the ZrS_3 electrode obtained from the equivalent circuit model (**Figure S7**c) at various stages of long-term cycling (refer to **Figure 5**d) under 3000 mA g^{-1} (OCV conditions). The table summarizes the fitted values of the equivalent circuit elements, including electrolyte/electrode interface resistances (R_s, R_{SEI}, R_{ct}, R_4), and the χ^2 fitting parameter. Refer to **Figure S7** for corresponding EIS spectra.

Electrodes	Equivalent circuit	R_s (Ω)	R_{SEI} (Ω)	R_{ct} (Ω)	R_4 (Ω)	χ^2
1 cycle	$R_s(R_{SEI}CPE_1)(R_{ct}CPE_2)(R_4CPE_3)W_s$	2.965	12.14	34.71	51.98	1.2×10^{-4}
10 cycles	$R_s(R_{SEI}CPE_1)(R_{ct}CPE_2)(R_4CPE_3)W_s$	2.11	10.83	43.84	57.05	9.1×10^{-5}
100 cycles	$R_s(R_{SEI}CPE_1)(R_{ct}CPE_2)(R_4CPE_3)W_s$	2.435	10.53	32.76	69.14	9.4×10^{-5}
1000 cycles	$R_s(R_{SEI}CPE_1)(R_{ct}CPE_2) (R_4CPE_3)W_s$	7.563	17	84.02	46.7	1.3×10^{-4}
3000 cycles	$R_s(R_{SEI}CPE_1)(R_{ct}CPE_2) (R_4CPE_3)W_s$	32.72	53.41	21.36	127	8.5×10^{-6}

At elevated temperature (60 °C), the ZrS_3 electrode exhibited rapid capacity fading, dropping from 398 mAh g^{-1} to ~63 mAh g^{-1} over 600 cycles, mainly due to accelerated SEI growth and parasitic reactions. In contrast, at room temperature (25 °C), the initial capacity was 354 mAh g^{-1} which gradually stabilized at ~290 mAh g^{-1}, indicating that thermal effects play an important role in long-term degradation under high-rate conditions (see **Figure S8**). In addition, the Coulombic efficiency (CE) of ZrS_3 at 25 °C stabilized rapidly after the initial cycles, maintaining values above 99.8% during long-term cycling, indicating good charge/discharge reversibility. In contrast, CE at 60 °C exhibited irregular fluctuations and occasional values exceeding 100%, which could be attributed to side reactions and progressive structural degradation during high-temperature operation.

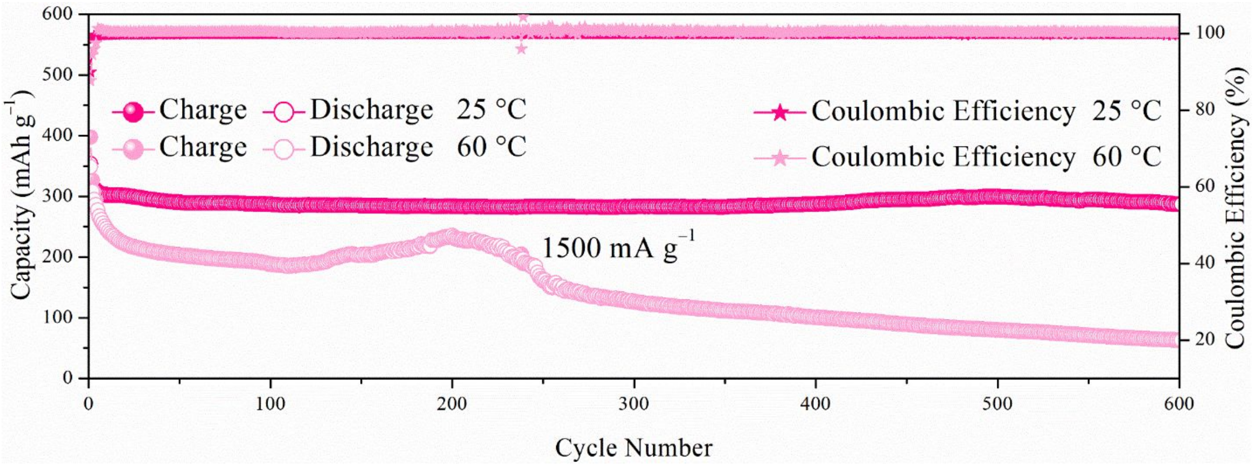

Figure S8. Cycling stability of ZrS_3 electrodes at 25 °C and 60 °C under a current density of 1500 mA g^{-1}.

Reference

(1) Chen, M.; Li, L.; Xu, M.; Li, W.; Zheng, L.; Wang, X. Quasi-One-Dimensional van Der Waals Transition Metal Trichalcogenides. *Research* **2023**, *6*. https://doi.org/10.34133/research.0066.

(2) Kang, J.; Sahin, H.; Peeters, F. M. Mechanical Properties of Monolayer Sulphides: A Comparative Study between MoS_2 , HfS_2 and TiS_3. *Physical Chemistry Chemical Physics* **2015**, *17* (41), 27742–27749. https://doi.org/10.1039/C5CP04576B.

(3) Mortazavi, B.; Shojaei, F.; Yagmurcukardes, M.; Makaremi, M.; Zhuang, X. A Theoretical Investigation on the Physical Properties of Zirconium Trichalcogenides, ZrS_3, $ZrSe_3$ and $ZrTe_3$ Monolayers. *Energies* **2022**, *15* (15), 5479. https://doi.org/10.3390/en15155479.

(4) Li, M.; Dai, J.; Zeng, X. C. Tuning the Electronic Properties of Transition-Metal Trichalcogenides via Tensile Strain. *Nanoscale* **2015**, *7* (37), 15385–15391. https://doi.org/10.1039/c5nr04505c.

(5) Conejeros, S.; Guster, B.; Alemany, P.; Pouget, J. P.; Canadell, E. Rich Polymorphism of Layered NbS_3. *Chemistry of Materials* **2021**, *33* (14), 5449–5463. https://doi.org/10.1021/acs.chemmater.1c01417.

(6) Sun, G.; Wei, Z.; Chen, N.; Chen, G.; Wang, C.; Du, F. Quasi-1D TiS_3: A Potential Anode for High-Performance Sodium-Ion Storage. *Chemical Engineering Journal* **2020**, *388* (15), 124305. https://doi.org/10.1016/j.cej.2020.124305.

(7) Bespalov, I.; Datler, M.; Buhr, S.; Drachsel, W.; Rupprechter, G.; Suchorski, Y. Initial Stages of Oxide Formation on the Zr Surface at Low Oxygen Pressure: An in Situ FIM and XPS Study. *Ultramicroscopy* **2015**, *159*, 147–151.

https://doi.org/10.1016/j.ultramic.2015.02.016.

(8) Xu, K. Electrolytes and Interphases in Li-Ion Batteries and Beyond. *Chemical Reviews* **2014**, *114* (23), 11503–11618. https://doi.org/10.1021/cr500003w.

(9) Zhang, S. S. A Review on Electrolyte Additives for Lithium-Ion Batteries. *Journal of Power Sources* **2006**, *162* (2), 1379–1394. https://doi.org/10.1016/j.jpowsour.2006.07.074.

(10) Nie, M.; Abraham, D. P.; Chen, Y.; Bose, A.; Lucht, B. L. Silicon Solid Electrolyte Interphase (SEI) of Lithium Ion Battery Characterized by Microscopy and Spectroscopy. *Journal of Physical Chemistry C* **2013**, *117* (26), 13403–13412. https://doi.org/10.1021/jp404155y.

(11) Verma, P.; Maire, P.; Novák, P. A Review of the Features and Analyses of the Solid Electrolyte Interphase in Li-Ion Batteries. *Electrochimica Acta* **2010**, *55* (22), 6332–6341. https://doi.org/10.1016/j.electacta.2010.05.072.

(12) Pang, H.; Sun, W.; Lv, L. P.; Jin, F.; Wang, Y. MOF-Templated Nanorice-Nanosheet Core-Satellite Iron Dichalcogenides by Heterogeneous Sulfuration for High-Performance Lithium Ion Batteries. *Journal of Materials Chemistry A* **2016**, *4* (48), 19179–19188. https://doi.org/10.1039/c6ta09060e.

(13) Sun, W.; Tao, X.; Du, P.; Wang, Y. Carbon-Coated Mixed-Metal Sulfide Hierarchical Structure: MOF-Derived Synthesis and Lithium-Storage Performances. *Chemical Engineering Journal* **2019**, *366* (15), 622–630. https://doi.org/10.1016/j.cej.2019.01.178.

(14) Wu, K.; Cao, X.; Li, M.; Lei, B.; Zhan, J.; Wu, M. Bottom-Up Synthesis of MoS_2/CNTs Hollow Polyhedron with 1T/2H Hybrid Phase for Superior Potassium-Ion Storage. *Small* **2020**, *16* (43), 2004178. https://doi.org/10.1002/smll.202004178.
